# Effects of experimental canopy openness on wood-inhabiting fungal fruiting diversity across succession

**DOI:** 10.1038/s41598-024-67216-1

**Published:** 2024-07-12

**Authors:** Jasper Schreiber, Petr Baldrian, Vendula Brabcová, Roland Brandl, Harald Kellner, Jörg Müller, Friederike Roy, Claus Bässler, Franz-Sebastian Krah

**Affiliations:** 1 Faculty of Biological Sciences, Institute for Ecology, Evolution and Diversity, Conservation Biology, Goethe University Frankfurt, 60438 Frankfurt am Main, Germany; 2 Laboratory of Environmental Microbiology, Institute of Microbiology of the Czech Academy of Sciences, 14200 Prague, Czech Republic; 3 Faculty of Biology, Department of Ecology, Animal Ecology, Philips University of Marburg, 35032 Marburg, Germany; 4 International Institute Zittau, Department of Bio- and Environmental Sciences, Technical University Dresden, 02763 Zittau, Germany; 5 Field Station Fabrikschleichach, Department of Animal Ecology and Tropical Biology Biocenter, University of Würzburg, 96181 Rauhenebrach, Germany; 6 Fungal Ecology and BayCEER, University of Bayreuth, Universitätsstr. 30, 95440 Bayreuth, Germany; 7 Bavarian Forest National Park, Grafenau, Germany; 8 Global Change Research Institute of the Czech Academy of Sciences, 603 00 Brno, Czech Republic

**Keywords:** Succession, Microclimate, Canopy mortality, Climate change, Fungi, Dead wood, Forest management, Biodiversity, Community ecology, Forest ecology

## Abstract

While the succession of terrestrial plant communities is well studied, less is known about succession on dead wood, especially how it is affected by environmental factors. While temperate forests face increasing canopy mortality, which causes considerable changes in microclimates, it remains unclear how canopy openness affects fungal succession. Here, we used a large real-world experiment to study the effect of closed and opened canopy on treatment-based alpha and beta fungal fruiting diversity. We found increasing diversity in early and decreasing diversity at later stages of succession under both canopies, with a stronger decrease under open canopies. However, the slopes of the diversity versus time relationships did not differ significantly between canopy treatments. The community dissimilarity remained mainly stable between canopies at ca. 25% of species exclusively associated with either canopy treatment. Species exclusive in either canopy treatment showed very low number of occupied objects compared to species occurring in both treatments. Our study showed that canopy loss subtly affected fungal fruiting succession on dead wood, suggesting that most species in the local species pool are specialized or can tolerate variable conditions. Our study indicates that the fruiting of the fungal community on dead wood is resilient against the predicted increase in canopy loss in temperate forests.

## Introduction

Forests are increasingly subjected to canopy mortality due to disturbances, forest management and climate change^1–3^. Especially disturbances and droughts increase canopy mortality, and the death of trees leads to the aggregation of dead wood if not removed due to pest control^4^. Open canopies lead to increased sun exposure and higher variability of temperature and moisture and thus create a variable environment, whereas microclimates below closed canopies buffer sun exposure and create more constant thermal and moisture conditions^5–7^. Further, forest canopies likely exceed 30 years to recover to pre-disturbance conditions such as canopy closure, which can even take longer if repeated disturbances cause recurring canopy loss^2,8, 9^. Since dead wood decays over years to decades^10^, depending on the size, organisms may face increasingly open canopies and associated changes in microclimate throughout the successional stages of wood-inhabiting organisms. Although several studies have shown that open canopy conditions affect various species groups’ richness and community composition on dead wood^11–15^, we have only a limited understanding of the succession of wood-inhabiting organisms in general and under contrasting canopy conditions, hampering predictions under future conditions such as increasing canopy loss^16^.

Fruit body-forming fungi are among the most diverse colonizers of dead wood, besides insects such as bark beetles^17–19^. Several studies have shown significant effects of canopy cover or canopy openness on wood-inhabiting fungal diversity^13,14, 20, 21^. For example, a real-world experiment found that the richness of wood-inhabiting fungi was non-significantly higher under closed compared with open canopies. Further, the composition differed significantly between closed and open canopies for two tree species^13^. Such previous studies mainly investigated the first years and stages of decay^13,22^, did not explicitly analyze succession^23^, focused on a subset of the fungal taxonomic breadth^24^, or surveyed different decay stages simultaneously of different dead wood items instead of following succession on the same items^25–29^. Therefore, we know little about the succession of fungal species on standardized dead wood objects in different microclimates. Studies investigating succession found a peak of species diversity at intermediate decay stages for fruit body data^30^ and at final decay stages for metabarcoding data^30,31^. Further, the succession was affected by the species composition of the initial colonizers causing priority effects^32^. Since wood-inhabiting fungi undergo a succession of species during decay^29^, one might expect differences in successional patterns with time between forest microclimate conditions^33,34^. Since moisture and temperature are important factors affecting fungal growth, canopy openness can be expected to affect species richness and community composition across successional stages^13,14, 35–40^. For example, wood-inhabiting species were shown to be differentially tolerant to temperature and moisture^15,41^. If species that colonize at different time points are differentially tolerant to microclimates, we would expect differences in the successional pattern of richness and composition. Based on these prior results, we hypothesize (i) increasing difference in alpha diversity due to lowering species numbers under open canopies if fewer species can tolerate variable microclimatic conditions (ii) increasing difference in community dissimilarity from early and late stages of decay because of species reduction under open canopies.

**Figure 1:**
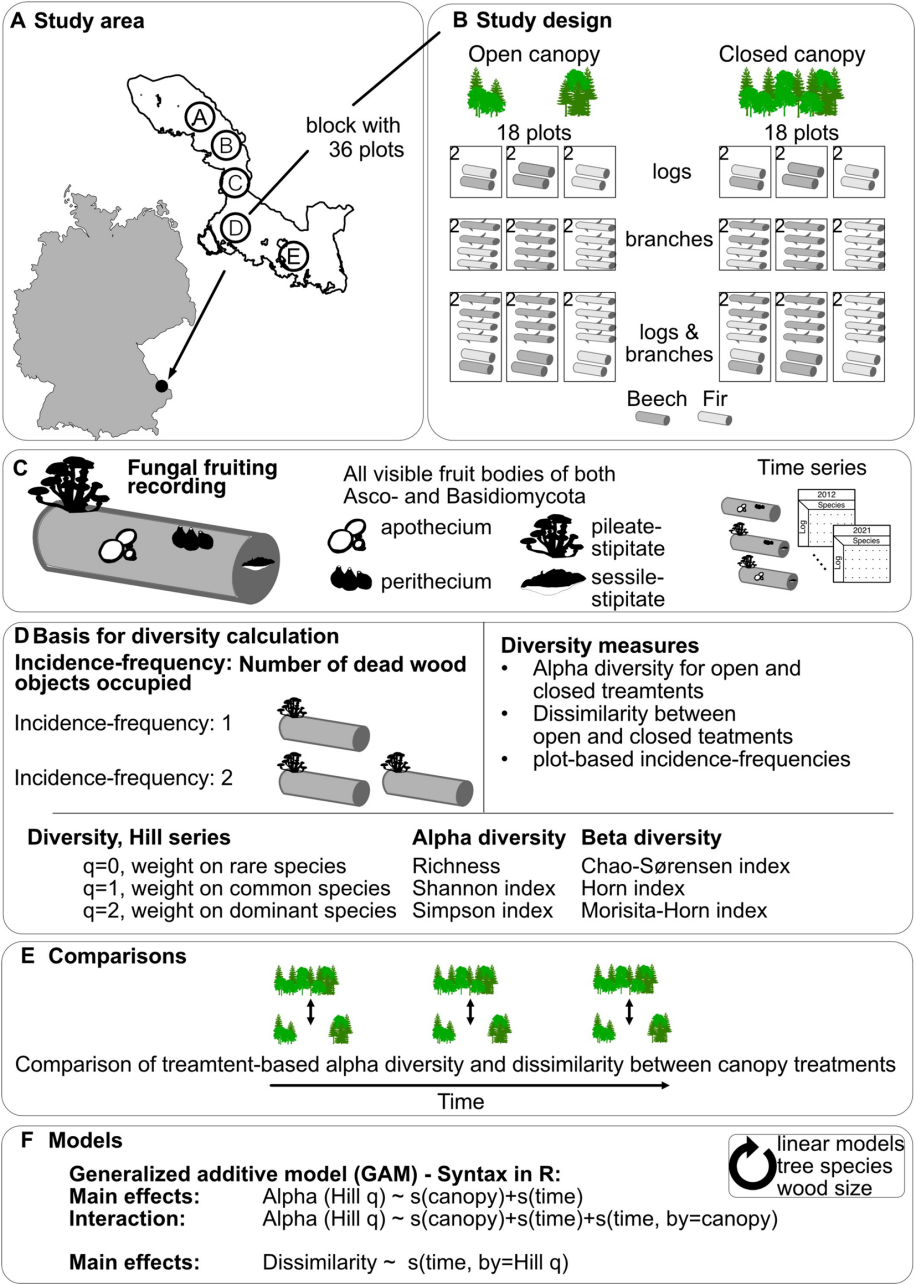
Conceptual graphic of the study design and analysis workflow. (**A**) The location of the National Park Bavarian Forest in the southeast of Germany with the alphabetical denotation showing the 5 random blocks of the dead wood experiment. (**B**) Within each block 18 plots are closed canopy stands and 18 result from 0.1 ha clearings of woody plants and yearly mowing. Within each plot, logs and/or branches of Beech and/or Fir were installed. (**C**) All visible fungal specimens were identified based on reproductive structures (fruit bodies) on all dead wood objects across a time series of 10 consecutive years. (**D**) Based on the dead wood logs occupied we calculated the incidence-based frequencies per species, i.e., the number of occupied objects. Alpha and beta diversity were compared by considering the importance of the frequency of species along the Hill series. (**E**) We used the incidence-based frequencies to compare alpha and beta diversity between closed and open canopy conditions across succession. (**F**) We used generalized additive models (GAMs) and linear models (LMs) to test the effect of time on alpha diversity in interaction with the canopy treatment and the effect of time on the dissimilarity with time by Hill numbers.

To address these hypotheses, we established a dead wood experiment with 240 dead wood logs and 480 dead wood branches of two host tree species (*Fagus sylvatica,* hereafter “beech”, and *Abies alba*, hereafter “fir”) half of them were exposed to closed canopies, while the other half to open canopies (Fig. 1A,B). Visible fungal species of Asco- and Basidiomycota were identified on each dead wood log and branch for 10 consecutive years (Fig. 1C). Canopies were kept open, and ground vegetation was mowed yearly to disentangle the effects of canopy-mediated microclimatic conditions from other edaphic factors affecting fungal performance during succession. We calculated treatment-based alpha and beta diversity (Fig. 1D) and compared them across the successional years (Fig. 1E). We further weighted the importance of species by their incidence frequency (number of dead wood objects occupied) to emphasize rare, common and dominant species along the Hill series (Fig. 1F)^42^.

## Material and methods

### Study area and study design

The dead-wood experiment was established in the management zone of the Bavarian Forest National Park in southeastern Germany in 2011 (Fig. 1A). The management zone is characterized by a mixed mountain forest with Norway spruces (*Picea abies* (L.) H. Karst), European beech (*Fagus sylvatica* L.) and Silver fir (*Abies alba* Mill.)^43^. Mean annual temperature in this zone is ca. 7 °C and mean sum of annual precipitation ca. 1300 mm^44^.

We created 36 plots with a size of 0.1 ha, which we replicated in 5 blocks across the landscape scale (Fig. 1A). Half of the plots were created under closed canopies (18) and half under open canopies (18) (Fig. 1B). The open canopies resulted from removing all living and dead trees from the plots. Annual mowing kept the plots open. Within the 18 plots, different combinations of dead wood tree species and dead wood types were designed to capture differences in dead-wood heterogeneity on the plot level. The combinations are shown within a schematic (Fig. 1B) and are described in detail in Refs. ^12,13^. In summary, across all five blocks and both canopy treatments, a total of 240 logs (5 blocks * 2 tree species * 24 logs) and 480 branches (5 blocks * 2 tree species * 48 branches) were placed on the forest floor. Seven dead wood objects could not be used for analysis because the objects themselves (branches) or their labels could not be found (stolen or broken off). The logs had a mean diameter of + /− SD = 33 + /− 6.5 cm, and a standardized length of 5 m. The branches had a mean diameter of + /− SD = 3.2 + /− 1.3 cm, and a mean length + /− SD = 2.7 + /− 0.88 m. The logs and branches were from stands of similar elevation, tree species composition, age, and size and were harvested with chainsaws and brought to the plots at the end of 2011.

Previous studies showed considerable and significant dead wood object surface temperature differences of ca. 20 °C on average for a summer day between open and closed canopies within the same experiment^21,45^. We measured wood surface temperatures on the upper surface of 136 logs, with each of five measures for every meter (segment) of the log (half under open, half under closed plots across the four blocks) in August 2018 using an infrared thermal sensor on a summer day. We found great differences in Light Detection and Ranging (LiDAR) penetration rate and temperature between open and closed canopies (Fig. S1). The LiDAR penetration rate is a reliable measure of radiation availability near the ground^46^.

### Fruit body inventories

We identified species based on fruit bodies that were visible with the naked eye on dead wood objects every autumn (September/October), the main season of fruit body development (Fig. 1C)^47^. To ensure an effective and non-redundant sampling, we divided logs into seven segments, each 1 m long. Two segments represented the log's cut edges, and five represented the log surface. The branches were considered as a single segment. Fruit bodies were identified in the field or, if necessary, in the laboratory with the aid of a microscope by mycological professionals (see *Acknowledgements*). Voucher specimens were deposited in the herbarium of the Bavarian Forest National Park. The nomenclature followed MycoBank^48^ and a complete species list is available in the Supplement (Table S1). During each field campaign, we estimated the stage of decomposition for each segment of the objects in four categories following Albrecht (1990)^49^. We then calculated the average decay stage per log based on the segments. Branches were treated as one segment. After 10 years, logs and branches were found in various decay stages, and some were already beginning to disintegrate (Figs. S1, S2).

### Data preparation

To address our research questions, we calculated alpha and beta diversity (Fig. 1D). We used the incidence-frequency as basis, defined as the number of dead wood objects occupied by a species. We computed this measure for each canopy treatment and combinations of the tree species and dead-wood size. We calculated the incidence-frequency across blocks and also for each block separately. The data for each block were limited for some treatments. Still, the observed patterns were consistent with the overall approach (data not shown) even when spatial autocorrelation was included. We, therefore, present the results of the analyses across blocks here.

To address our research question, we additionally applied an approach based on Hill numbers^50^. The Hill framework allows the calculation of diversity indices based on the Hill number *q*, which gives increasing weight to the species frequencies^42^ and has been used intensively for community ecological research^51–53^. q = 0 weights infrequent species based on incidence data and has been termed as “rare species”, q = 1 weights frequently detected species and has been termed as “common species” and, q = 2 weights highly frequent species and has been termed as “dominant species”.

To calculate the estimated alpha diversity of fungal fruiting communities, we used the function *iNEXT* within the R package 'iNEXT'^54^. This package provides diversity estimates based on rarefaction and extrapolation using incidence-frequencies. We used species richness (q = 0), the Shannon diversity index (q = 1)^55^, and the Simpson diversity index (q = 2)^56^. We used dissimilarities as beta diversity measure. To analyze the dissimilarities between communities, we first estimated species similarity indices between open and closed canopies for each tree species and year using the *SimilarityPair* function within the R package 'SpadeR'^57^. To consider different similarity indices, we calculated the similarities of three Hill numbers: the Chao-Sørensen index (q = 0), the Horn index (q = 1), and the Morisita-Horn index (q = 2). Second, we calculated 1—estimated similarity values, to gain the dissimilarity of communities. The dissimilarity values range from 0 (equal communities) and 1 (completely different communities, meaning no shared species). Each estimate includes the 95% confidence interval based on 100 bootstraps. For a schematic overview of the variables used, please see Fig. 1D.

### Statistical analysis

#### Alpha diversity

To test if treatment-based alpha diversity responded differently under closed and open canopies over time, we used generalized additive models (GAM, R package 'mgcv' function *gam*^58^). We used this approach to capture potential non-linear responses, which can be expected due to species accumulation^13,59^. Further, we did not include interactions between canopy and tree species, as we regarded tree species as replicates of the study. We additionally fit linear models (LMs). For an overview of the models fit, see Fig. 1F.

We first fit a model with the alpha diversity measures (q along the Hill series) as the response variable and factorial canopy openness and continuous time (years) as predictor variables. We used fewer knots (k = 3) for the smooth term, because the model did not converge with default values. Within a second GAM, we included an interaction term between time and canopy treatment to test for significantly divergent patterns between closed and open canopies over time. This can be achieved by a second GAM, which is specified as the first and adding the term “s(time, by = canopy)”, which then gives a single estimate of the interaction term. We repeated this model for each tree species, wood size and Hill number (q) separately. A significant interaction would indicate differing slopes between closed and open canopies over time. However, a significant interaction can only inform of different slopes, not if single years are significantly different. Therefore, we also interpreted non-overlapping 95% confidence intervals as trends of differences between canopy treatments each year. Note that non-overlapping confidence intervals do not necessitate significance, so we interpret trends cautiously. We also used linear models (LM, R package 'stats' function *lm*), because many trends showed linear behavior and because testing interaction terms using LMs is more established than using GAMs^60^. We first fit a linear model with canopy treatment as a factor and time as a continuous predictor and a second model adding the interaction term of canopy and time. In both cases, GAM and LM, we used the fixed effects from the first model and the interaction term from the second model. We additionally inspected autocorrelation plots (R package ‘stats’ function *acf*) of the model residuals and did not observe signs of temporal autocorrelation, indicating no substantial influence of autocorrelation. Interpreting *p*-values from the first model with fixed effects and the second model with interaction effects would violate statistical principles, e.g., testing identical response and predictor variables in two models. Since our main interest was on the marginal effect of the canopy with time interaction, we only interpreted p-values for the interaction term.

#### Beta diversity

To test if dissimilarity between treatments changed over time, we used GAMs. We used dissimilarity as the response variable continuous time as the predictor variable. We further added the Hill series *q* as a grouping factor. We repeated the models for each tree species and wood size. The overall dissimilarity range of the slope was interpreted as the dissimilarity between closed and open canopies, with low dissimilarity for values < 0.5. Furthermore, we calculated a linear model and a posthoc test, using the R package 'stats' function *TukeyHSD*, to test if the pairwise dissimilarity means differ between dissimilarity estimates of rare, common and dominant species. Where necessary, we applied Bonferroni corrections on the interpretation of p-values by considering significant only those below 0.016 due to multiple testing along the Hill series with three levels (i.e., 0.05/3).

To gain a further deeper understanding, (i) we reported overall species numbers associated uniquely with closed and open canopies to better understand differences in beta diversity between microclimate treatments. (ii) We showed the top two species that occurred either exclusively under closed or opened canopies or were found under both canopies. We also show their incidence-frequency over time. (iii) We prepared a table with the species exclusively found in either treatment and in time windows of two years to better understand the species that are most relevant for each treatment.

### Ethics declarations

All national guidelines on species protection as well as the IUCN Declaration on Research on Endangered Species and the Convention on Trade in Endangered Species of Wild Fauna and Flora were complied within this study.

## Results

**Figure 2:**
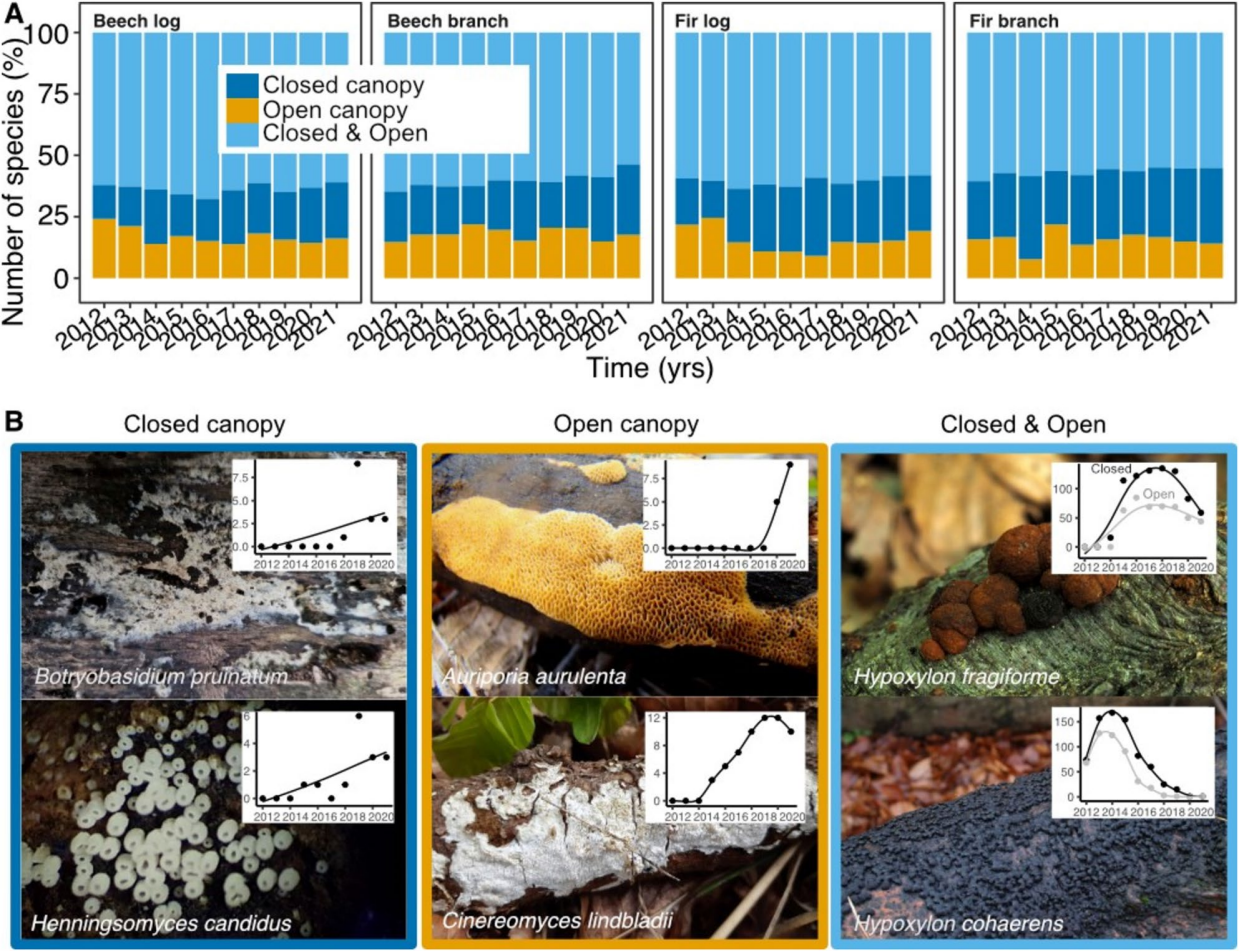
(**A**) The scaled number of species found shared and exclusively under the canopy treatments. (**B**) The most frequent two species occurring exclusively under closed, exclusively under open canopies and under both. Image credentials: *H. fragiforme* (Beech), *H. cohaerens* (Beech) and *H. candidus* (Fir) by Peter Karasch; *B. pruinatum* (Beech & Fir) by Jacob Heilmann-Clausen; *A. aurulenta* (Beech & Fir) by Joseba Castillo Munsuri and *C. lindbladii* (Beech & Fir) by gfiebes, both from iNaturalist. All images were used unaltered and were provided under CC BY-NC 4.0. Insets are incidence-frequencies over time with GAM smooth splines.

We found 486 different fungal fruiting species across all dead wood logs and branches over a timespan of 10 years. On beech, we found 387 and on fir 307 species. On dead wood logs, we found 417 and on branches 304 species. In closed canopies, we found 397 species and under open canopies 363 species. Across years, we found up to ca. 25% of species uniquely in open, up to ca. 25% of species uniquely in closed canopies and more than ca. 50% in both microclimate treatments (Fig. 2). The species exclusive to either treatment are shown in Table 1.

**Table 1:** Species exclusively found within closed and open treatments and in time windows of two years.

Canopy treatment	Occurrence exclusively in years			
	1–2	3–4	5–6	7–8
	Species	Species	Species	Species
Species exclusively fruited under open canopies	*Hymenoscyphus conscriptus*	*Ceriporiopsis gilvescens*	*Bisporella subpallida*	*Capitotricha bicolor*
	*Lachnellula subtilissima*	*Hypholoma marginatum*	*Capitotricha fagiseda*	*Dasyscypha nivea*
	*Peniophora violaceolivida*	*Hypochnicium wakefieldiae*	*Capronia pulcherrima*	*Galzinia incrustans*
	*Pezicula cinnamomea*	*Phanerochaete filamentosa*	*Durella macrospora*	*Hygrophoropsis aurantiaca*
	*Phanerochaete raduloides*	*Pluteus semibulbosus*	*Echinosphaeria canescens*	*Hypocrea protopulvinata*
	*Sistotrema confluens*	*Stereum rameale*	*Hyaloscypha britannica*	*Ischnoderma resinosum*
	*Tylospora asterophora*		*Hypochnicium geogenium*	*Kneiffiella barba-jovis*
	*Valsaria insitiva*		*Hypomyces aurantius*	*Lentomitella cirrhosa*
			*Junghuhnia nitida*	*Phlebia queletii*
			*Phellinus ferruginosus*	*Postia guttulata*
			*Trechispora minima*	*Psilocybe phyllogena*
			*Tulasnella inclusa*	*Sistotrema sernanderi*
				*Thelephora atra*
				*Trametes multicolor*
				*Trechispora microspora*
				*Trechispora nivea*
				*Tremella obscura*
				*Trichophaea pseudogregaria*
Species exclusively fruited under closed canopies	*Asterosporium hoffmannii*	*Auricularia auricula-judae*	*Colacogloea peniophorae*	*Athelia arachnoidea*
	*Bulgaria inquinans*	*Basidioradulum radula*	*Crepidotus versutus*	*Basidiodendron eyrei*
	*Byssomerulius corium*	*Flammulaster carpophilus*	*Entoloma cetratum*	*Botryobasidium aureum*
	*Calycina discreta*	*Hohenbuehelia atrocoerulea*	*Exidiopsis calcea*	*Byssocorticium caeruleum*
	*Hyalorbilia berberidis*	*Hohenbuehelia pinacearum*	*Flagelloscypha minutissima*	*Chaetosphaeria fusiformis*
	*Hymenoscyphus scutula*	*Melanomma sanguinarium*	*Herpotrichia macrotricha*	*Eriosphaeria aggregata*
	*Sistotrema coroniferum*	*Nectria cosmariospora*	*Hymenochaete cruenta*	*Flavophlebia sulfureoisabellina*
	*Sistotrema efibulatum*	*Olla scropulosa*	*Hyphodiscus hymeniophila*	*Gloeophyllum odoratum*
		*Phaeohelotium trabinellum*	*Kneiffiella microspora*	*Helminthosphaeria odontiae*
		*Phlebia acerina*	*Lachnum impudicum*	*Lachnum fasciculare*
		*Unguicularia cirrhata*	*Mycena zephirus*	*Leptosporomyces galzinii*
			*Tulasnella pinicola*	*Mycena metata*
			*Tulasnella thelephorea*	*Orbilia leucostigma*
				*Pholiota limonella*
				*Psathyrella obtusata*
				*Pseudotomentella umbrina*
				*Simocybe coniophora*

**Figure 3:**
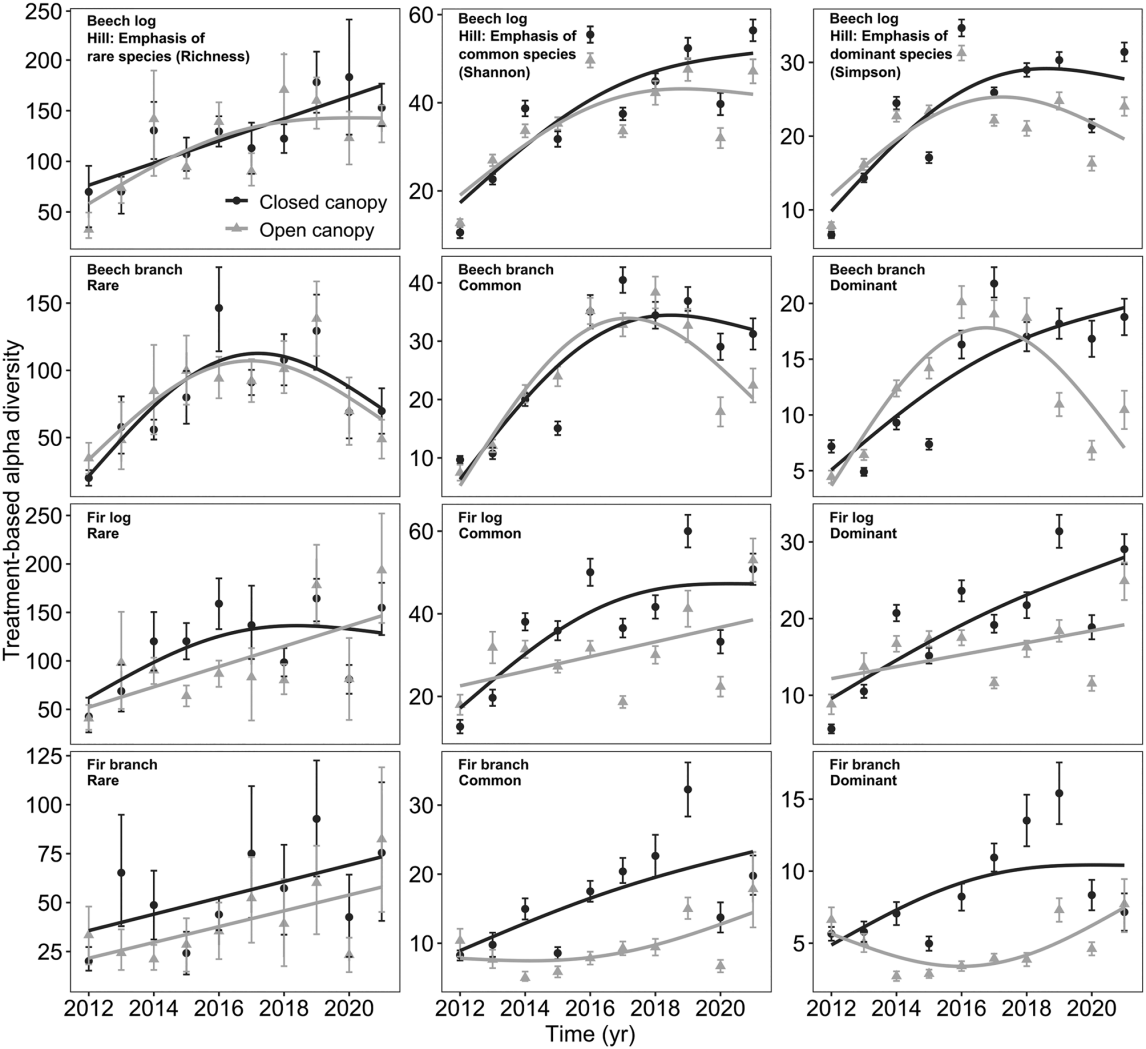
Treatment-based alpha diversity of fungal fruiting communities under closed (black) and open (grey) canopy treatments with time (years). Smooth splines are based on generalized additive models. Error bars are the 95% confidence intervals. For statistics, see Table 2.

We found an increasing treatment-based alpha diversity with time for logs and branches of both tree species (Fig. 3, Tables 2, 3). We did not find significant interactions between time and microclimate treatment (Tables 2, 3). However, temporal responses of fungal diversity differed when response trends and confidence intervals were considered: treatment-based alpha diversity of common and dominant species showed similar trends in the first years but diverging trends in later years between microclimate treatments; in later years, common and dominant species showed lower alpha diversity trends in open canopies (Fig. 3). The responses were stronger for communities on fir than beech. Treatment-based alpha diversity of rare species showed almost non-distinguishable trends between closed and open canopies, with mainly overlapping confidence intervals (Fig. 3).

**Table 2:** Statistics table for treatment-based alpha diversity in response to time and the canopy treatment for dead wood logs.

			General additive model (GAM)					Linear model		
			Fixed	Smooth terms						
Tree	q	Predictor	t	edf	F	p	R2	t	p	R2
Beech	0 rare	Intercept	15.07				0.54	− 4.72		0.53
		Canopy - open vs. closed	− 0.78					− 0.75		
		Time		1.60	15.73			4.75		
		Time x Canopy		2.04	1.39	0.290		− 0.35	0.729	
	1 common	Intercept	15.81				0.56	− 4.42		0.49
		Canopy - open vs. closed	− 0.84					− 0.72		
		Time		1.86	17.96			4.45		
		Time x Canopy		1.00	1.17	0.298		− 0.86	0.404	
	2 dominant	Intercept	14.60				0.63	− 2.81		0.26
		Canopy - open vs. closed	− 1.12					− 0.89		
		Time		1.91	10.87			2.83		
		Time x Canopy		1.00	2.69	0.123		− 1.14	0.270	
Fir	0 rare	Intercept	9.94				0.44	− 3.14		0.32
		Canopy - open vs. closed	− 0.94					− 0.94		
		Time		1.00	9.96			3.16		
		Time x Canopy		1.00	0.27	0.608		0.52	0.608	
	1 common	Intercept	11.88				0.32	− 3.23		0.37
		Canopy - open vs. closed	− 1.62					− 1.62		
		Time		1.00	10.62			3.26		
		Time x Canopy		1.00	0.99	0.334		− 1.00	0.334	
	2 dominant	Intercept	12.72				0.37	− 3.71		0.44
		Canopy- open vs. closed	− 1.79					− 1.79		
		Time		1.00	13.93			3.73		
		Time x Canopy		1.00	3.15	0.095		− 1.77	0.095	

We fit generalized additive models (GAM) and linear models. “Time x Canopy” denotes an interaction term. The alpha level is 0.016 (Bonferroni adjustment due to multiple comparisons). P values in brackets were not interpreted due to repeated testing. The abbreviations stand for: t = t-value, edf = effective degrees of freedom, F = F-value, *p* = *p*-value, R2 = R-squared.

**Table 3:** Statistics table for treatment-based alpha diversity in response to time and the canopy treatment for dead wood branches.

			General additive model (GAM)					Linear model		
			Fixed	Smooth terms						
Tree	q	Predictor	t	edf	F	p	R2	t	p	R2
Beech	0 rare	Intercept	12.74				0.63	− 1.74		0.05
		Canopy - open vs. closed	− 0.17					− 0.11		
		Time		1.96	17.20			1.76		
		Time x Canopy		1.00	0.50	0.490		− 0.43	0.670	
	1 common	Intercept	15.58				0.75	− 3.32		0.33
		Canopy - open vs. closed	− 0.79					− 0.49		
		Time		1.97	28.04			3.34		
		Time x Canopy		1.51	3.02	0.162		− 0.87	0.396	
	2 dominant	Intercept	11.16				0.53	− 2.51		0.20
		Canopy - open vs. closed	− 0.81					− 0.62		
		Time		1.92	10.62			2.53		
		Time x Canopy		3.03	**8.23**	**0.005**		− 1.65	0.119	
Fir	0 rare	Intercept	9.68				0.35	− 2.94		0.35
		Canopy - open vs. closed	− 1.86					− 1.86		
		Time		1.00	8.79			2.96		
		Time x Canopy		1.00	0.00	0.959		− 0.05	0.959	
	1 common	Intercept	10.67				0.48	− 2.99		0.48
		Canopy - open vs. closed	− 3.27					− 3.27		
		Time		1.00	9.06			3.01		
		Time x Canopy		1.00	1.24	0.283		− 1.11	0.283	
	2 dominant	Intercept	10.70				0.42	− 2.02		0.42
		Canopy - open vs. closed	− 3.39					− 3.39		
		Time		1.00	4.17			2.04		
		Time x Canopy		3.26	**6.14**	**0.012**		− 1.03	0.318	

We fit generalized additive models (GAM) and linear models. “Time x Canopy” denotes an interaction term. The alpha level is 0.016 (Bonferroni adjustment due to multiple comparisons). P values for fixed effects are not displayed due to repeated testing. The abbreviations stand for: t = t-value, edf = effective degrees of freedom, F = F-value, *p* = *p*-value, R2 = R-squared.

Significant values are in [bold].

Communities on fir showed lower treatment-based alpha diversity in open compared to closed canopies throughout the succession, a trend which increased over time for common and dominant and remained constant for rare species (Fig. 3).

**Figure 4:**
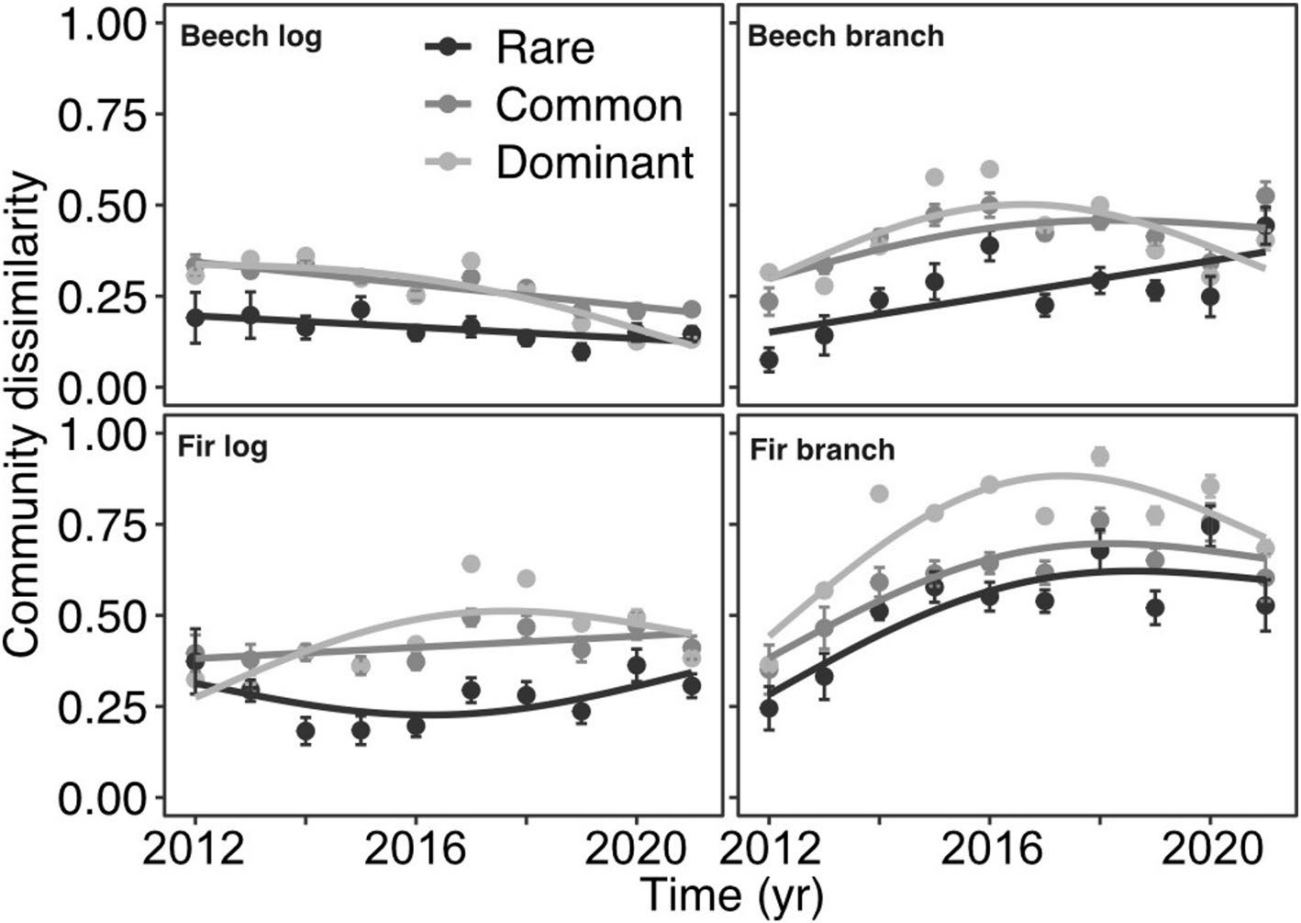
Treatment-based community dissimilarity of fungal communities between canopies with time (years). Smooth splines are based on generalized additive models. Error bars are the 95% confidence intervals. For statistics, see Table 3.

We found dissimilarity values of ca. 0.25 between microclimates on logs of both tree species and branches of beech (Fig. 4) and mostly non-significant trends of dissimilarity with time (Fig. 4, Table 4). We found higher dissimilarity values on fir branches, mainly for dominant and common species (Fig. 4). The average dissimilarity of rare species was significantly lower than that of common and dominant species (Fig. 4, Table 4).

**Table 4:** Statistics table for treatment-based community dissimilarity in response to time.

Predictor	Tree	q	edf	F	p	Tukey pairwise means			
						R2	Pair	t	p
Time (yr)	Beech log	0, rare	1.00	1.24	0.275	0.37	0–1	**4.09**	**0.001**
		1, common	1.00	4.76	0.038		0–2	**3.60**	**0.003**
		2, dominant	1.46	**8.37**	**0.005**		1–2	− 0.49	0.878
	Fir log	0, rare	1.53	0.47	0.662	0.05	0–1	**3.99**	**0.001**
		1, common	1.00	0.48	0.494		0–2	**4.66**	** < 0.001**
		2, dominant	2.20	2.01	0.110		1–2	0.67	0.782
Time (yr)	Beech branch	0, rare	1.00	1.24	0.275	0.37	0–1	**3.29**	**0.007**
		1, common	1.00	4.76	0.038		0–2	**3.44**	**0.005**
		2, dominant	1.46	**8.37**	**0.005**		1–2	0.15	0.988
	Fir branch	0, rare	1.54	3.07	0.048	0.05	0–1	1.26	0.428
		1, common	1.45	2.25	0.089		0–2	**3.36**	**0.006**
		2, dominant	2.14	3.90	0.030		1–2	2.10	0.109

We fit generalized additive models (GAM) and linear models. Using the Tukey posthoc test we tested for pairwise differences in means between diversity measures. The alpha level is 0.016 (Bonferroni adjustment due to multiple comparisons). The abbreviations stand for: t = t-value, edf = effective degrees of freedom, F = F-value, *p* = p-value, R2 = R-squared.

Significant values are in [bold].

## Discussion

In this study, we used experimentally opened vs. closed forest canopies to contrast fungal treatment-based alpha and beta fruiting diversity across 10 years of dead-wood succession. Successional treatment-based alpha diversity patterns were mostly similar, with a trend towards lower richness under open canopies in later successional stages. We found moderate differences in dissimilarity between microclimate treatments that persisted over succession with up to ca. 25% of species uniquely associated with either microclimate treatment.

We found a trend towards lower richness in later years between the microclimate treatments, however, interaction terms were not significant. Further, we found that confidence intervals did not overlap in many cases, supporting the trend towards lower richness under open canopies in later years (Fig. 3). Thus, we did not find clear support for our first hypothesis but a trend following this expectation. Comparable studies using fungal treatment-based alpha diversity between canopy conditions are scarce, especially in a time series context. However, one study of the initial decay stage (first four years), using six tree species, found no significant alpha diversity differences between microclimate treatments among tree species^14^. The authors found no apparent differences in alpha diversity between canopy treatments for five of six tree species, including beech and fir. We also found no strong differences in the early decay stage. However, we found a trend towards differences in later years. This effect might become more robust and even more advanced in the decay stages in the following years. Thus, long time series under standardized conditions are necessary to illuminate diversity patterns.

One explanation for lower richness in later years might be that fruiting was reduced by the microclimatic conditions under open canopies, e.g., due to higher maximum temperatures and thus increased water loss, preventing from forming fruit bodies. Fungal fruiting is largely driven by macroclimate^38^ and meteorological factors^61–64^ often following a phase with high precipitation in summer and a cooling in fall^65^ and can be delayed by drought^66^. With climate warming, fruiting, therefore, was observed to change towards longer fruiting seasons^65^. However, we have very limited knowledge available on the effects of experimental treatments on fruiting in real-world experiments. In the early phase, the same experiment as studied here, found that the species fruited under open conditions had tougher fruit bodies, likely to reduce water loss^45^. Adding to this, we here found only one species (*Hypholoma marginatum*) exclusively under open conditions that formed soft-fleshed mushrooms (pileate-stipitate fruit body). Further, this species fruited in the year 3–4, but no species exclusively under open conditions had such soft-fleshed fruiting bodies in later years (Table 1). In contrast, under closed conditions, we found five species that also fruited in later years such as *Entoloma cetratum*, *Mycena zephirus*, *Psathyrella obtusata*, *Pholiota limonella*, and *Simocybe coniophora* (Table 1). Thus, the fluctuating conditions under open canopies seem to be limiting for some species that produce fruit bodies with high water demand. However, further analyses are necessary to understand the adaptations and cues of fungal fruiting under variable microclimates. Another explanation might be that the species are lost from the substrates as mycelium due to unfavorable growth conditions and cannot be recorded in the fruiting record. We cannot disentangle these two explanations. This could be approached by estimating the presence of mycelium of species by metabarcoding or metatranscriptomes. Indeed, a series of publications have investigated metabarcoding of wood samples vs. fruit body-based sampling. How well above-ground reproductive structures in fungi can inform about below-ground mycelium is not entirely certain, especially across environmental gradients. Species prevalent with a high number of reads based on environmental sequencing approaches in deadwood also produced many fruit bodies^30^. However, this study also showed that some highly abundant species detected via sequencing could only rarely be found in the fruit body record. In another study, some rare fungal species could only be detected with fruit body sampling but not via environmental samples and metabarcoding^67^. Therefore, both approaches have limitations, and ideally, both are used jointly^68^. Nevertheless, fruit bodies are formed only when physiological and nutrient conditions of the mycelium and environmental conditions are suitable^69^. The fruiting community is likely a subset of the species growing as vegetative mycelium, and it will be interesting in future studies to better understand why and under which conditions some produce fruit bodies and others do not. Nevertheless, the trend towards species loss or fruiting reduction in later years may indicate a change in successional trajectories.

However, even if the open canopy microclimate conditions are harsh and lead to a reduction of species, it is unclear why this would only happen in the later decay stages. One explanation might be based on the dominance-tolerance trade-off^39^ and the characteristics of secondary colonizers. First, according to the dominance-tolerance trade-off, wood-inhabiting fungi either have narrow environmental tolerance but strong competitive abilities or vice versa. Second, first and secondary colonizers (those colonizing after endophytic fungi) require strong competitive abilities^70^ and, therefore, might have low tolerance towards microclimatically fluctuating conditions. Taken together, colonizers in later decay stages may require increasingly competitive abilities to outcompete established species and may then have difficulties growing under open canopy conditions, which are characterized by variable conditions due to narrow tolerance. To test this hypothesis, manipulated fungal communities are required under steady vs. variable edaphic conditions.

It is a long-standing question whether environmental fluctuations lead to an increase or decrease in diversity^71,72^. Rapoport’s rule, for example, predicts higher species ranges in northern areas^73^, and one explanation is that species are more adapted to intense seasonality and thus are more tolerant towards changing conditions or fluctuations on smaller temporal scales as well. Indeed, a study on global soil fungi showed that species ranges are larger towards higher latitudes^36^. For fungi, a higher phylogenetic diversity was further found where thermal seasonality is stronger across Europe^74^. Across organism groups, fluctuating conditions in environmental variables can lead to variable responses, ranging from a decrease in diversity for planktons^75^, increased diversity for bacteria^76^ and increased diversity of wood-inhabiting fungi^77^. In later decay stages, we found a tendency towards lower diversity under open canopies (more fluctuating conditions) but not in early decay stages. A previous study used 16 fungal isolates within microcosms for 6 months and found an increase in species richness with increasing temperature fluctuation and suggested niche differentiation of the species as a mechanism for greater coexistence^77^. Thus, our results are partly in contrast with previous knowledge. The tendency towards lower diversity in later succession indicates that the effect of fluctuating microclimate differs across the succession and, thus, the number and composition of species. Therefore, to better understand the effects of environmental fluctuations based on canopy openness, an experiment could test how established communities react to sudden change from benign to fluctuating conditions, e.g., by community establishment under closed canopies with subsequent transfer to open conditions replicated across different decay stages. Further, our results from the temperate region might not apply to climates with more constant conditions, such as in humid tropics, and thus, replication of our experiment in such biomes would be important.

Our analyses further revealed that alpha diversity responded more strongly when common and dominant species were emphasized than when rare species were emphasized (Fig. 3). Thus, our results suggest that rare species may be more tolerant towards microclimatic fluctuations across succession. The Rapoports’s rule might thus only apply to rare species. We also considered different tree species and wood sizes in our experiment. We found stronger divergence of common and dominant species in alpha diversity with time between canopies in branches than logs (Fig. 3). Two explanations may be possible: (i) a previous study found that logs and branches harbor significantly different fungal fruiting communities when standardized for the surface area^13^, which may respond more sensitive to open canopy conditions. (ii) The decomposition is faster within branches, and thus, the decay stage reached is already more advanced (Fig. S2), and later communities react stronger than earlier communities. Since we saw an increasing difference in alpha diversity with time within the logs it is reasonable to extrapolate further in time that effects may become more strongly in even later decay stages. Therefore, we hypothesize that communities associated with the third or fourth decay stage may be more sensitive to microclimatic fluctuations.

One alternative explanation for the reduced alpha diversity under open canopies may be differences in decay rates (successional speed) or moisture contents between dead wood in closed vs. open canopies. The decay under open canopies was found to be faster than under closed canopies^78–81^. However, one paper found no differences in the decomposition after 10 years of decay of logs between microclimates^23^. Thus we have inconclusive evidence whether decomposition increases, decreases or remains stable within different microclimates. Further, during decomposition the moisture content of dead wood increased^82,83^, however, this was not studied yet in different microclimates. Nevertheless, differences in diversity between canopies might reflect differences in decay rates or moisture changes rather than succession (time). If true, we would expect no apparent differences in diversity patterns between canopies, when decay is the predictor instead of time. We found non-significant interactions between the decay stage and canopy treatment on alpha diversity (Fig. S3, Table S2) and no significant trends in beta diversity (Fig. S4, Table S3). However, although the interaction terms of the models were non-significant, we found non-overlapping confidence intervals and curve trends into different directions between microclimates for some years (Fig. S3). These results suggest either little differences in moisture content or decay stages between treatments or that species are little affected by them.

Finally, our study also contains limitations. First, the absence of a fruiting record cannot inform whether a species can grow as mycelium, but the presence provides information on which species can reproduce successfully. Second, we did not measure decay rates directly (e.g., mass loss^84^), but used a visual classification of dead wood objects using a four-classes system. We chose this approach because measuring decay rates via weighing is a destructive method and thus may disrupt succession. Further, visual decay classes are able to detect coarse differences in decomposition variation^85,86^. Third, our study does not capture the full successional trajectory. However, we cover the 10 years on the same objects, of which some reached decay stages three and four within this time frame (Fig. S2).

In conclusion, it is predicted that forest canopies will become increasingly opened up, and thus, potentially harsher microclimates will become more frequent^3^. Across succession, more fluctuating environments under open canopies only slightly affected species alpha diversity. However, we observed a trend towards a reduction of alpha diversity of common and dominant species in later decay stages. Under increasingly open conditions, late-decay fungi may experience difficulties in the future, which may also affect decomposition and humidification rates. Further, we found one-fourth of species fruiting exclusively under open canopies, and thus, open-canopy specialists may increase in population size. In summary, our study indicates that the fruiting of the fungal fruiting community on dead wood is currently rather resilient against the microclimatic fluctuations associated with the open canopy habitat and the predicted increase in canopy loss in temperate forests.

## Supplementary Information

Supplementary Information.

## Data Availability

The generated dataset that support the findings of this study is openly available in FIGSHARE https://figshare.com/s/7ace58d986dd8c8ff94f.
