# Correction: Effects of experimental canopy openness on wood-inhabiting fungal fruiting diversity across succession

**DOI:** 10.1038/s41598-025-27944-4

**Published:** 2025-12-01

**Authors:** Jasper Schreiber, Petr Baldrian, Vendula Brabcová, Roland Brandl, Harald Kellner, Jörg Müller, Friederike Roy, Claus Bässler, Franz‑Sebastian Krah

**Affiliations:** 1 Faculty of Biological Sciences, Institute for Ecology, Evolution and Diversity, Conservation Biology, Goethe University Frankfurt, 60438 Frankfurt am Main, Germany; 2 Laboratory of Environmental Microbiology, Institute of Microbiology of the Czech Academy of Sciences, 14200 Prague, Czech Republic; 3 Faculty of Biology, Department of Ecology, Animal Ecology, Philips University of Marburg, 35032 Marburg, Germany; 4 International Institute Zittau, Department of Bio‑ and Environmental Sciences, Technical University Dresden, 02763 Zittau, Germany; 5 Field Station Fabrikschleichach, Department of Animal Ecology and Tropical Biology Biocenter, University of Würzburg, 96181 Rauhenebrach, Germany; 6 Fungal Ecology and BayCEER, University of Bayreuth, Universitätsstr. 30, 95440 Bayreuth, Germany; 7 Bavarian Forest National Park, Grafenau, Germany; 8 Global Change Research Institute of the Czech Academy of Sciences, 603 00 Brno, Czech Republic

Correction to: *Scientific Reports* 10.1038/s41598-024-67216-1, published online 12 July 2024

The original version of this Article contained errors.

Due to an error during the labelling of the ‘treatment-based alpha diversity’ variable, the group labels for ‘common’, ‘dominant’ and ‘rare’ were inadvertently given as ‘rare’, ‘common’ and ‘dominant’, respectively.

Consequently, in the Results section,

“However, temporal responses of fungal diversity differed when response trends and confidence intervals were considered: treatment-based alpha diversity of rare and common species showed similar trends in the first years but diverging trends in later years between microclimate treatments; in later years, rare and common species showed lower alpha diversity trends in open canopies (Fig. 3). The responses were stronger for communities on fir than beech. Treatment-based alpha diversity of dominant species showed almost non-distinguishable trends between closed and open canopies, with mainly overlapping confidence intervals (Fig. 3).

Communities on fir showed lower treatment-based alpha diversity in open compared to closed canopies throughout the succession, a trend which increased over time for rare and common and remained constant for dominant species (Fig. 3).”

now reads:

“However, temporal responses of fungal diversity differed when response trends and confidence intervals were considered: treatment-based alpha diversity of common and dominant species showed similar trends in the first years but diverging trends in later years between microclimate treatments; in later years, common and dominant species showed lower alpha diversity trends in open canopies (Fig. 3). The responses were stronger for communities on fir than beech. Treatment-based alpha diversity of rare species showed almost non-distinguishable trends between closed and open canopies, with mainly overlapping confidence intervals (Fig. 3)

Communities on fir showed lower treatment-based alpha diversity in open compared to closed canopies throughout the succession, a trend which increased over time for common and dominant and remained constant for rare species (Fig. 3).”

In addition, in the Discussion section,

“Our analyses further revealed that alpha diversity responded more strongly when rare and common species were emphasized than when dominant species were emphasized (Fig. 2). Thus, our results suggest that dominant species may be more tolerant towards microclimatic fluctuations across succession. The Rapoports’s rule might thus only apply to dominant species. We also considered different tree species and wood sizes in our experiment. We found stronger divergence of rare and common species in alpha diversity with time between canopies in branches than logs (Fig. 2).”

now reads:

“Our analyses further revealed that alpha diversity responded more strongly when common and dominant species were emphasized than when rare species were emphasized (Fig. 3). Thus, our results suggest that rare species may be more tolerant towards microclimatic fluctuations across succession. The Rapoports’s rule might thus only apply to rare species. We also considered different tree species and wood sizes in our experiment. We found stronger divergence of common and dominant species in alpha diversity with time between canopies in branches than logs (Fig. 3).”

And,

“However, we observed a trend towards a reduction of alpha diversity of rare and common species in later decay stages.”

now reads:

“However, we observed a trend towards a reduction of alpha diversity of common and dominant species in later decay stages.”

Finally, the labels were incorrect in Figure 3, Table 2, Table 3, Supplementary Figure 3 and Supplementary Table 2.

The original Fig. 3 and accompanying legend appear below.

**Fig. 3:**
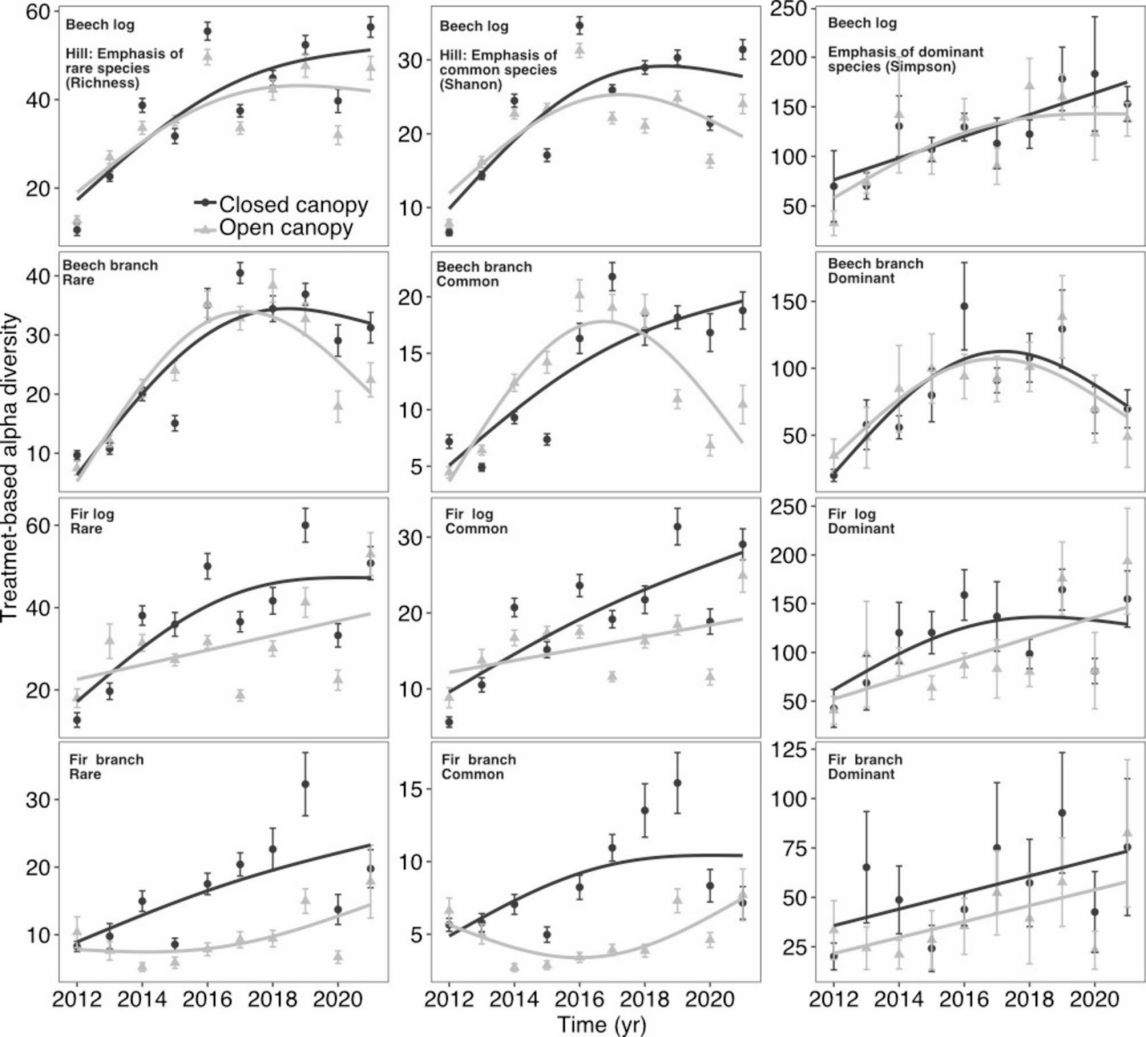
Treatment-based alpha diversity of fungal fruiting communities under closed (black) and open (grey) canopy treatments with time (years). Smooth splines are based on generalized additive models. Error bars are the 95% confidence intervals. For statistics, see Table 2.

The original Table 2 and accompanying legend appear below.

**Table 2:** Statistics table for treatment-based alpha diversity in response to time and the canopy treatment for dead wood logs.

Tree	q	Predictor	General additive model (GAM)	Smooth terms				Linear model		
			Fixed	Smooth terms			R2			
			t	edf	F	p		t	p	R2
Beech	0 rare	Intercept	15.81				0.56	− 4.42		0.49
		Canopy—open vs. closed	− 0.84					− 0.72		
		Time		1.86	17.96			4.45		
		Time × Canopy		1.00	1.17	0.298		− 0.86	0.404	
	1 common	Intercept	14.60				0.63	− 2.81		0.26
		Canopy—open vs. closed	− 1.12					− 0.89		
		Time		1.91	10.87			2.83		
		Time × Canopy		1.00	2.69	0.123		− 1.14	0.270	
	2 dominant	Intercept	15.07				0.54	− 4.72		0.53
		Canopy—open vs. closed	− 0.78					− 0.75		
		Time		1.60	15.73			4.75		
		Time × Canopy		2.04	1.39	0.290		− 0.35	0.729	
Fir	0 rare	Intercept	11.88				0.32	− 3.23		0.37
		Canopy—open vs. closed	− 1.62					− 1.62		
		Time		1.00	10.62			3.26		
		Time × Canopy		1.00	0.99	0.334		− 1.00	0.334	
	1 common	Intercept	12.72				0.37	− 3.71		0.44
		Canopy- open vs. closed	− 1.79					− 1.79		
		Time		1.00	13.93			3.73		
		Time × Canopy		1.00	3.15	0.095		− 1.77	0.095	
	2 dominant	Intercept	9.94				0.44	− 3.14		0.32
		Canopy—open vs. closed	− 0.94					− 0.94		
		Time		1.00	9.96			3.16		
		Time × Canopy		1.00	0.27	0.608		0.52	0.608	

We fit generalized additive models (GAM) and linear models. “Time x Canopy” denotes an interaction term. The alpha level is 0.016 (Bonferroni adjustment due to multiple comparisons). *P* values in brackets were not interpreted due to repeated testing. The abbreviations stand for: t = t-value, edf = effective degrees of freedom, F = F-value, *p* = *p*-value, R2 = R-squared.

The original Table 3 and accompanying legend appear below.

**Table 3:** Statistics table for treatment-based alpha diversity in response to time and the canopy treatment for dead wood branches.

Tree	q	Predictor	General additive model (GAM)					Linear model		
			Fixed	Smooth terms			R2			
			t	edf	F	p		t	p	R2
Beech	0 rare	Intercept	15.58				0.75	− 3.32		0.33
		Canopy—open vs. closed	− 0.79					− 0.49		
		Time		1.97	28.04			3.34		
		Time × Canopy		1.51	3.02	0.162		− 0.87	0.396	
	1 common	Intercept	11.16				0.53	− 2.51		0.20
		Canopy—open vs. closed	− 0.81					− 0.62		
		Time		1.92	10.62			2.53		
		Time × Canopy		3.03	**8.23**	**0.005**		− 1.65	0.119	
	2 dominant	Intercept	12.74				0.63	− 1.74		0.05
		Canopy—open vs. closed	− 0.17					− 0.11		
		Time		1.96	17.20			1.76		
		Time × Canopy		1.00	0.50	0.490		− 0.43	0.670	
Fir	0 rare	Intercept	10.67				0.48	− 2.99		0.48
		Canopy—open vs. closed	− 3.27					− 3.27		
		Time		1.00	9.06			3.01		
		Time × Canopy		1.00	1.24	0.283		− 1.11	0.283	
	1 common	Intercept	10.70				0.42	− 2.02		0.42
		Canopy—open vs. closed	− 3.39					− 3.39		
		Time		1.00	4.17			2.04		
		Time × Canopy		3.26	**6.14**	**0.012**		− 1.03	0.318	
	2 dominant	Intercept	9.68				0.35	− 2.94		0.35
		Canopy—open vs. closed	− 1.86					− 1.86		
		Time		1.00	8.79			2.96		
		Time × Canopy		1.00	0.00	0.959		− 0.05	0.959	

We fit generalized additive models (GAM) and linear models. “Time x Canopy” denotes an interaction term. The alpha level is 0.016 (Bonferroni adjustment due to multiple comparisons). *P* values for fixed effects are not displayed due to repeated testing. The abbreviations stand for: t = t-value, edf = effective degrees of freedom, F = F-value, *p* = *p*-value, R2 = R-squared. Significant values are in [bold].

The original Article and accompanying Supplementary Information file have been corrected.

