# Supplementary material for: Effects of experimental canopy openness on wood-inhabiting fungal fruiting diversity across succession
Source: Sci Rep. 2024 Jul 12;14:16135. doi: 10.1038/s41598-024-67216-1 (PMC11245472; doi:10.1038/s41598-024-67216-1)
Supplement: Supplementary file 1 — Supplementary Information. [file 41598_2024_67216_MOESM1_ESM.docx]

“Effects of experimental canopy openness on wood-inhabiting fungal fruiting diversity across succession”

**Supplementary Information**

**Figures**


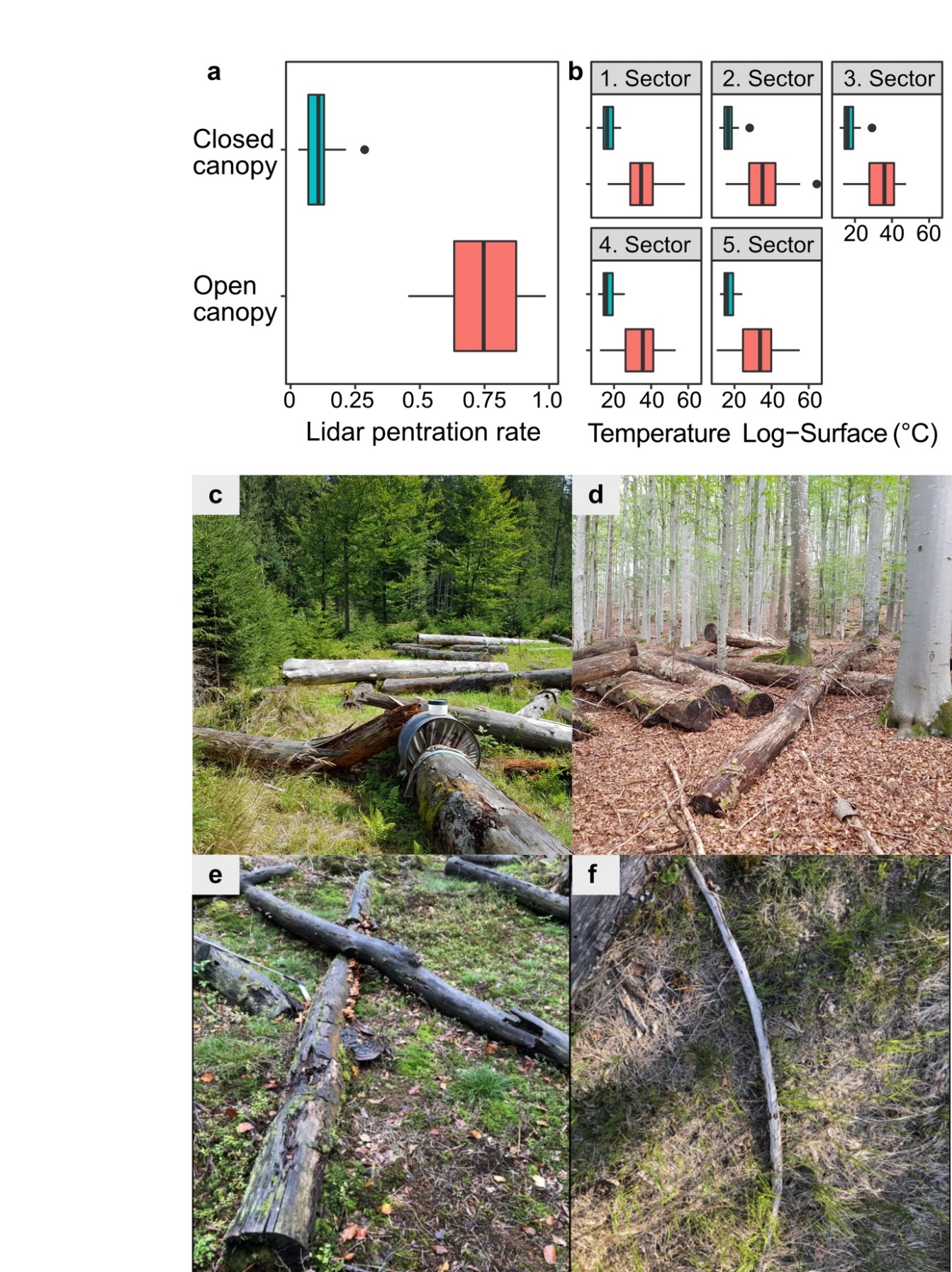


**Figure S1** Demonstration of the microclimatic differences between closed (blue) and open canopies (red), based on a) LiDAR penetration rate, b) temperature measured on the surface of dead-wood logs on the same sunny day on a subset of the logs in the experiment for each of the five sectors on the log. c) Exemplary representative image of dead wood logs under opened canopy treatment. Open canopy treatments are 0.1 ha clearings of all trees and shrubs. Further vegetation is mowed once per year to avoid shading by a dense grass layer surrounding the logs. On the image, this can be seen on the left side of the image border where a mowing edge is visible. d) Exemplary representative image of dead wood logs under the closed canopy treatment. e-f) Exemplary images of dead wood log and branch after 10 years of the experiment. Photos by Jasper Schreiber.

**
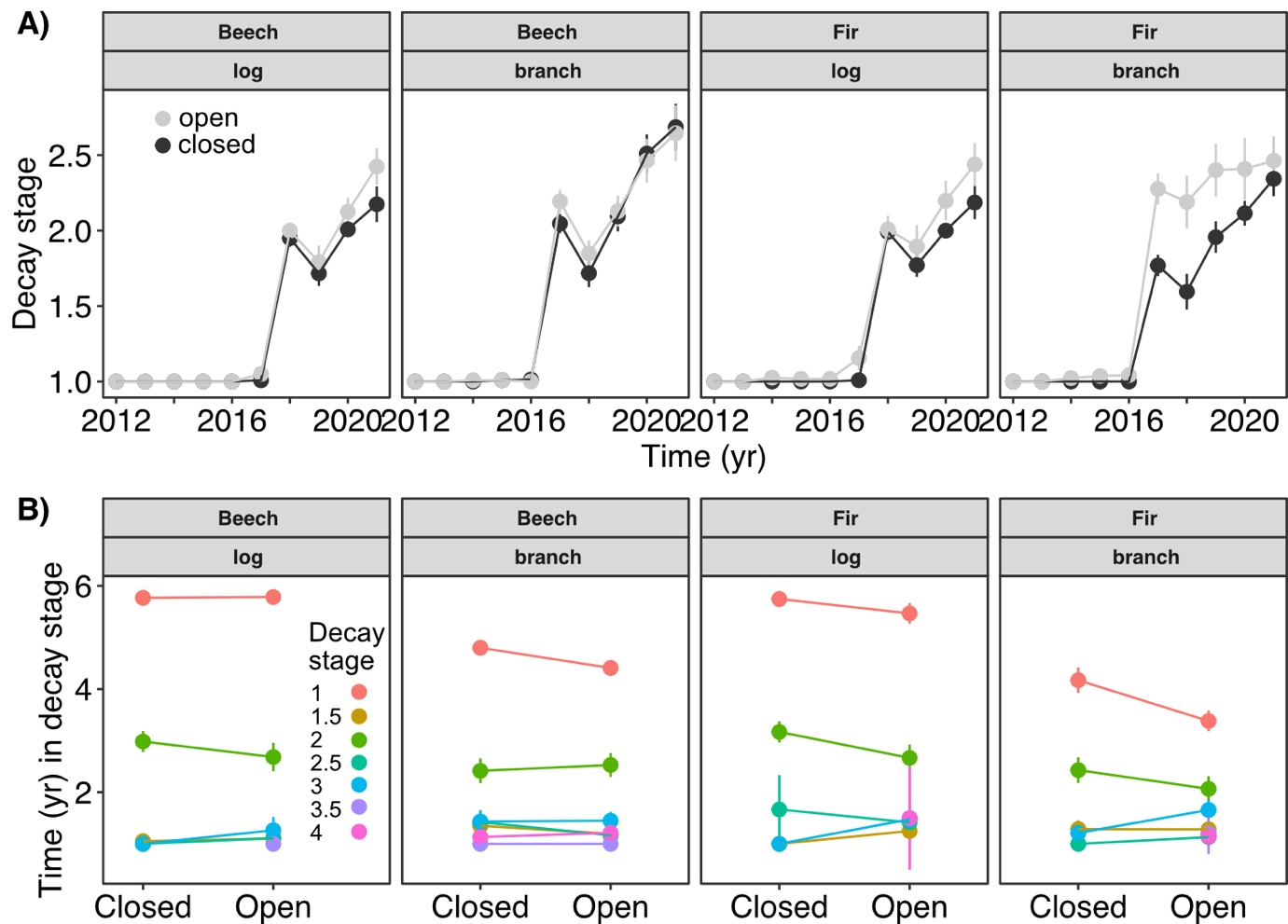
**

**Figure S2 Decay stages of dead wood objects.** A) Average decay stage of logs and branches in the years between canopy treatments. During each field campaign, we estimated the stage of decomposition for each segment of the objects (four categories according to (Albrecht 1990)). Each log was separated into seven segments. Each branch was treated as one sector. We then calculated the average decay stage per dead wood object based on the segments and then per year across objects. Please note the slight decrease in the decay stage in the year 2018 (branches) and 2019 (logs) is due to a different composition of recorders. B) Average time (years) a log or branch remains in a given decay stage. Data points are the means and error bars indicate double standard error.

**
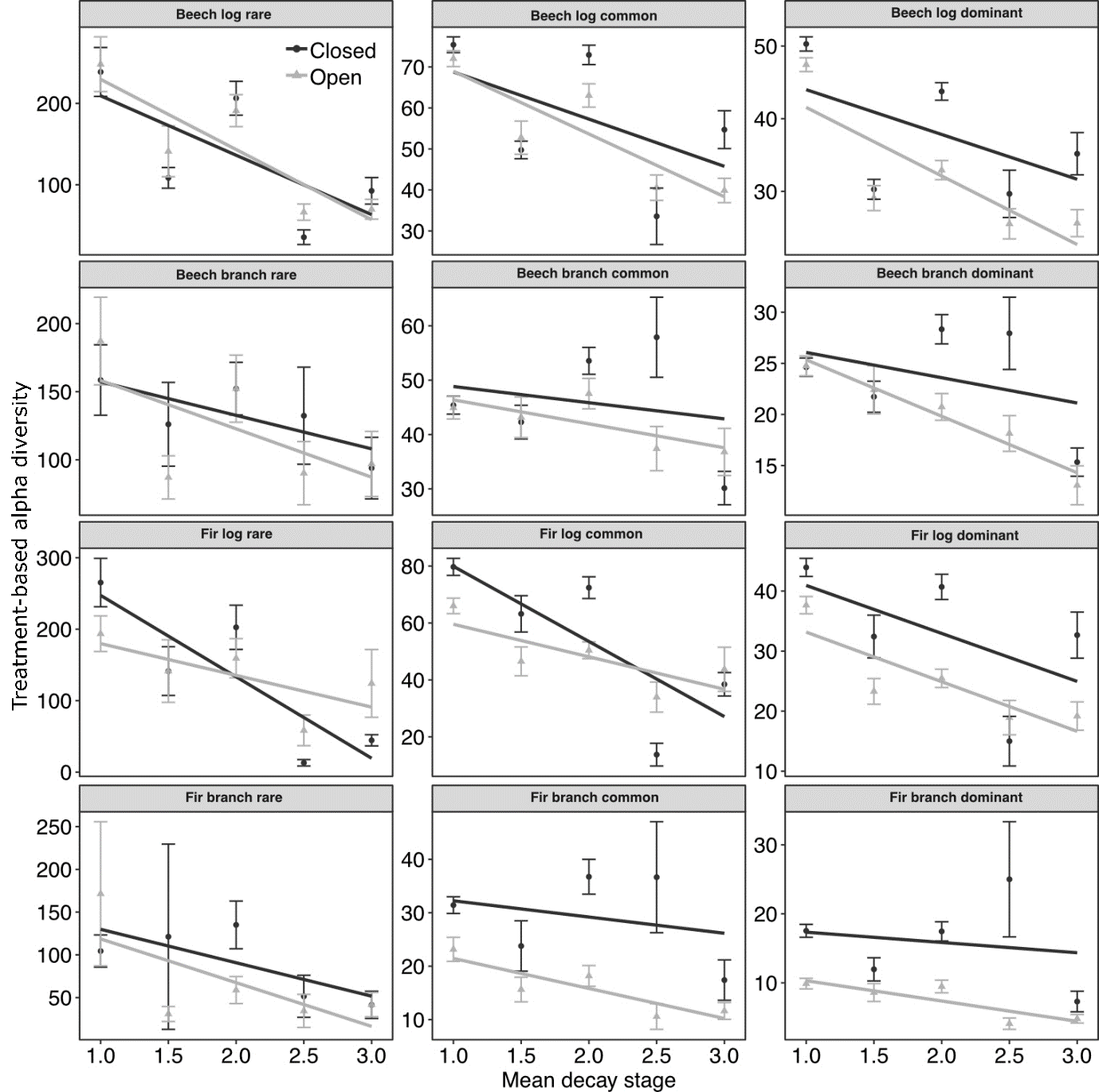
Figure S3 Treatment-based alpha diversity of fungal fruiting communities under closed (black) and open (grey) canopy treatments with decay stages.** The canopy treatments include closed and open canopy conditions for fungal growth. The estimated treatment-based alpha diversity based on incidence-frequencies of three rare, common and dominant species using Hill numbers. Regression curves are based on linear models with continuous decay stage as predictor and treatment-based alpha diversity as response variable. Error bars are the 95% confidence intervals. For statistics table see Table S2.


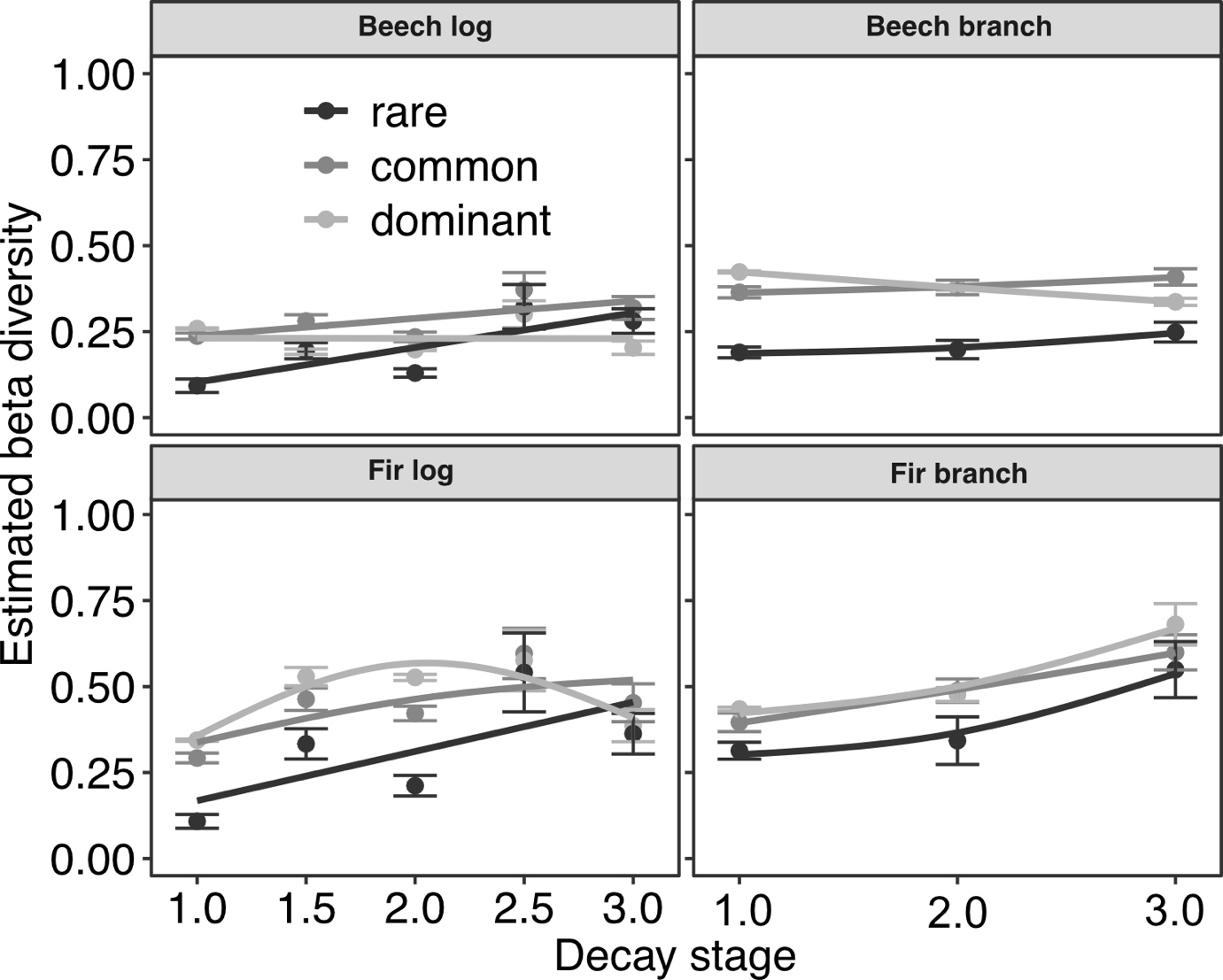


**Figure S4 Beta diversity (dissimilarity) of fungal communities between canopy treatments with decay stages.** The estimated beta diversity is based on incidence-frequencies of rare (q=0: Chao-Sørensen index, black line), common (q=1: Horn index, grey line) and dominant species (q=2: Morisita-Horn index, light grey line) using Hill numbers. Smooth splines are based on generalized additive models (GAM) with continuous decay stage as smoothed predictor and beta diversity as response variable. Error bars are the 95% confidence intervals.

**Tables**

**Table S1** List of fruiting species of fungi and the number of colonized dead wood objects across 10 years of the experiment.

| Species | Beech Closed branch | Beech Closed log | Beech Open branch | Beech Open log | Fir Closed branch | Fir Closed log | Fir Open branch | Fir Open log |
| --- | --- | --- | --- | --- | --- | --- | --- | --- |
| *Aleurodiscus amorphus* | 0 | 0 | 0 | 0 | 47 | 0 | 15 | 1 |
| *Alutaceodontia alutacea* | 0 | 0 | 1 | 0 | 0 | 1 | 0 | 2 |
| *Amphinema byssoides* | 0 | 0 | 0 | 1 | 1 | 0 | 0 | 2 |
| *Amylostereum chailletii* | 0 | 0 | 0 | 0 | 21 | 58 | 1 | 24 |
| *Antrodia heteromorpha* | 0 | 0 | 0 | 0 | 1 | 0 | 0 | 0 |
| *Antrodia serialis* | 0 | 0 | 0 | 2 | 0 | 1 | 0 | 3 |
| *Antrodia sinuosa* | 0 | 0 | 0 | 0 | 0 | 0 | 0 | 2 |
| *Antrodiella faginea* | 3 | 5 | 12 | 11 | 0 | 0 | 0 | 0 |
| *Antrodiella leucoxantha* | 0 | 0 | 0 | 1 | 0 | 0 | 0 | 0 |
| *Antrodiella onychoides* | 0 | 0 | 0 | 1 | 0 | 0 | 0 | 0 |
| *Antrodiella pallescens* | 0 | 0 | 0 | 1 | 0 | 0 | 0 | 0 |
| *Antrodiella romellii* | 0 | 0 | 1 | 0 | 0 | 0 | 0 | 0 |
| *Antrodiella semisupina* | 0 | 0 | 1 | 1 | 0 | 0 | 0 | 0 |
| *Antrodiella serpula* | 0 | 0 | 0 | 2 | 0 | 0 | 0 | 0 |
| *Aphanobasidium pseudotsugae* | 0 | 0 | 0 | 0 | 1 | 0 | 0 | 1 |
| *Armillaria gallica* | 0 | 3 | 0 | 0 | 0 | 0 | 0 | 1 |
| *Armillaria ostoyae* | 0 | 1 | 0 | 5 | 0 | 0 | 0 | 4 |
| *Ascocoryne cylichnium* | 0 | 17 | 1 | 14 | 0 | 4 | 0 | 2 |
| *Ascocoryne sarcoides* | 35 | 51 | 23 | 39 | 1 | 6 | 1 | 9 |
| *Ascocoryne solitaria* | 8 | 0 | 11 | 0 | 0 | 1 | 0 | 1 |
| *Ascodichaena rugosa* | 0 | 4 | 0 | 0 | 0 | 0 | 0 | 0 |
| *Ascotremella faginea* | 2 | 1 | 0 | 0 | 0 | 0 | 0 | 0 |
| *Asterosporium hoffmannii* | 0 | 2 | 0 | 0 | 0 | 0 | 0 | 0 |
| *Athelia arachnoidea* | 0 | 0 | 0 | 0 | 0 | 1 | 0 | 0 |
| *Athelia bombacina* | 0 | 0 | 0 | 0 | 0 | 1 | 1 | 1 |
| *Athelia decipiens* | 2 | 7 | 0 | 1 | 3 | 9 | 0 | 5 |
| *Athelia epiphylla* | 19 | 18 | 3 | 11 | 12 | 10 | 0 | 4 |
| *Athelia pyriformis* | 0 | 0 | 2 | 0 | 2 | 0 | 0 | 0 |
| *Auricularia auricula-judae* | 0 | 1 | 0 | 0 | 0 | 0 | 0 | 0 |
| *Auriporia aurulenta* | 0 | 0 | 0 | 3 | 0 | 0 | 0 | 9 |
| *Barbatosphaeria barbirostris* | 5 | 36 | 4 | 3 | 0 | 1 | 0 | 0 |
| *Basidiodendron caesiocinereum* | 0 | 1 | 1 | 0 | 0 | 1 | 0 | 0 |
| *Basidiodendron eyrei* | 0 | 1 | 0 | 0 | 0 | 2 | 0 | 0 |
| *Basidioradulum radula* | 0 | 0 | 0 | 0 | 0 | 1 | 0 | 0 |
| *Bertia latispora* | 0 | 0 | 1 | 0 | 10 | 19 | 2 | 1 |
| *Bertia moriformis* | 8 | 16 | 1 | 2 | 0 | 0 | 0 | 0 |
| *Biscogniauxia nummularia* | 0 | 1 | 0 | 0 | 0 | 0 | 0 | 0 |
| *Bispora antennata* | 5 | 55 | 4 | 51 | 0 | 5 | 0 | 2 |
| *Bisporella citrina* | 24 | 47 | 3 | 22 | 0 | 2 | 0 | 0 |
| *Bisporella pallescens* | 5 | 35 | 0 | 10 | 0 | 0 | 0 | 0 |
| *Bisporella subpallida* | 0 | 0 | 0 | 1 | 0 | 0 | 0 | 0 |
| *Bjerkandera adusta* | 0 | 47 | 0 | 45 | 0 | 11 | 1 | 35 |
| *Boidinia furfuracea* | 0 | 1 | 0 | 0 | 3 | 24 | 0 | 4 |
| *Botryobasidium aureum* | 2 | 0 | 0 | 0 | 0 | 0 | 0 | 0 |
| *Botryobasidium botryoideum* | 0 | 0 | 0 | 1 | 0 | 2 | 0 | 1 |
| *Botryobasidium botryosum* | 1 | 1 | 1 | 1 | 3 | 4 | 0 | 3 |
| *Botryobasidium candicans* | 0 | 1 | 2 | 2 | 6 | 5 | 0 | 6 |
| *Botryobasidium conspersum* | 3 | 8 | 2 | 4 | 0 | 0 | 0 | 2 |
| *Botryobasidium intertextum* | 1 | 0 | 0 | 0 | 0 | 0 | 0 | 2 |
| *Botryobasidium laeve* | 0 | 5 | 2 | 6 | 1 | 3 | 1 | 9 |
| *Botryobasidium medium* | 0 | 0 | 0 | 0 | 0 | 1 | 0 | 4 |
| *Botryobasidium obtusisporum* | 0 | 2 | 0 | 1 | 0 | 1 | 0 | 3 |
| *Botryobasidium pruinatum* | 3 | 6 | 0 | 0 | 0 | 2 | 0 | 0 |
| *Botryobasidium subcoronatum* | 15 | 19 | 2 | 10 | 9 | 29 | 0 | 11 |
| *Botryobasidium vagum* | 21 | 16 | 17 | 16 | 41 | 26 | 15 | 30 |
| *Botryohypochnus isabellinus* | 0 | 1 | 0 | 1 | 0 | 1 | 0 | 1 |
| *Brunnipila fuscescens* | 4 | 0 | 0 | 0 | 0 | 0 | 0 | 0 |
| *Bulgaria inquinans* | 0 | 1 | 0 | 0 | 0 | 0 | 0 | 0 |
| *Byssocorticium atrovirens* | 0 | 1 | 0 | 0 | 0 | 0 | 0 | 0 |
| *Byssocorticium caeruleum* | 1 | 0 | 0 | 0 | 0 | 0 | 0 | 0 |
| *Byssomerulius corium* | 1 | 0 | 0 | 0 | 0 | 0 | 0 | 0 |
| *Calocera cornea* | 32 | 58 | 39 | 57 | 2 | 2 | 0 | 2 |
| *Calocera furcata* | 0 | 1 | 0 | 0 | 22 | 21 | 4 | 33 |
| *Calocera viscosa* | 2 | 2 | 2 | 0 | 0 | 12 | 0 | 2 |
| *Calosphaeria pulchella* | 0 | 0 | 0 | 0 | 0 | 1 | 0 | 1 |
| *Calycina discreta* | 0 | 1 | 0 | 0 | 0 | 0 | 0 | 0 |
| *Calycina languida* | 0 | 7 | 0 | 0 | 0 | 1 | 0 | 0 |
| *Camarops lutea* | 0 | 1 | 0 | 0 | 0 | 0 | 0 | 0 |
| *Cantharellus tubaeformis* | 0 | 4 | 0 | 0 | 1 | 16 | 0 | 0 |
| *Capitotricha bicolor* | 0 | 0 | 4 | 0 | 0 | 0 | 0 | 0 |
| *Capitotricha fagiseda* | 0 | 0 | 2 | 0 | 0 | 0 | 0 | 0 |
| *Capronia pulcherrima* | 0 | 0 | 2 | 0 | 0 | 0 | 0 | 0 |
| *Catinella olivacea* | 0 | 1 | 0 | 0 | 0 | 0 | 0 | 0 |
| *Ceraceomyces eludens* | 0 | 0 | 0 | 0 | 0 | 1 | 0 | 0 |
| *Ceraceomyces serpens* | 2 | 4 | 0 | 3 | 2 | 1 | 0 | 0 |
| *Ceratobasidium cornigerum* | 0 | 1 | 0 | 0 | 0 | 2 | 0 | 2 |
| *Ceratostomella ampullasca* | 1 | 0 | 11 | 2 | 0 | 0 | 0 | 0 |
| *Ceratostomella rostrata* | 0 | 0 | 0 | 0 | 0 | 0 | 0 | 1 |
| *Ceriporia excelsa* | 0 | 2 | 2 | 0 | 0 | 0 | 0 | 0 |
| *Ceriporia viridans* | 1 | 1 | 0 | 2 | 0 | 0 | 0 | 0 |
| *Ceriporiopsis gilvescens* | 0 | 0 | 0 | 0 | 0 | 0 | 0 | 1 |
| *Chaetosphaeria fusiformis* | 0 | 0 | 0 | 0 | 1 | 0 | 0 | 0 |
| *Chaetosphaeria myriocarpa* | 1 | 1 | 1 | 0 | 0 | 3 | 1 | 0 |
| *Chaetosphaeria ovoidea* | 5 | 13 | 2 | 0 | 0 | 1 | 0 | 0 |
| *Chaetosphaeria pulviscula* | 2 | 0 | 1 | 0 | 1 | 0 | 0 | 0 |
| *Ciboria dumbirensis* | 1 | 0 | 0 | 1 | 0 | 0 | 0 | 0 |
| *Cinereomyces lindbladii* | 0 | 0 | 2 | 2 | 0 | 0 | 5 | 11 |
| *Cistella dentata* | 0 | 0 | 1 | 0 | 0 | 0 | 0 | 0 |
| *Claussenomyces atrovirens* | 2 | 5 | 0 | 1 | 0 | 1 | 0 | 0 |
| *Clitocybe ditopus* | 0 | 1 | 0 | 0 | 1 | 2 | 0 | 2 |
| *Clitopilus hobsonii* | 0 | 3 | 0 | 1 | 2 | 4 | 0 | 0 |
| *Colacogloea effusa* | 0 | 2 | 0 | 0 | 0 | 0 | 0 | 0 |
| *Colacogloea peniophorae* | 1 | 0 | 0 | 0 | 0 | 0 | 0 | 0 |
| *Coniochaeta spec.* | 2 | 5 | 12 | 25 | 1 | 5 | 6 | 14 |
| *Coniochaeta ligniaria* | 0 | 0 | 9 | 5 | 1 | 5 | 0 | 4 |
| *Coniochaeta malacotricha* | 0 | 0 | 5 | 1 | 6 | 6 | 7 | 18 |
| *Coniochaeta pulveracea* | 0 | 2 | 26 | 29 | 0 | 1 | 8 | 20 |
| *Coniochaeta subcorticalis* | 0 | 0 | 1 | 2 | 0 | 0 | 1 | 0 |
| *Coniochaeta velutina* | 0 | 1 | 62 | 46 | 0 | 4 | 4 | 16 |
| *Coniophora arida* | 0 | 1 | 0 | 1 | 0 | 0 | 6 | 1 |
| *Coniophora olivacea* | 0 | 0 | 0 | 0 | 0 | 2 | 1 | 3 |
| *Coprinus laanii* | 0 | 0 | 0 | 0 | 0 | 2 | 0 | 0 |
| *Coprinus micaceus* | 0 | 1 | 0 | 4 | 0 | 0 | 0 | 0 |
| *Coriolopsis gallica* | 0 | 0 | 0 | 2 | 0 | 0 | 0 | 0 |
| *Creopus gelatinosus* | 1 | 0 | 0 | 0 | 0 | 0 | 0 | 1 |
| *Crepidotus applanatus* | 0 | 0 | 0 | 0 | 0 | 0 | 0 | 1 |
| *Crepidotus cesatii* | 1 | 0 | 0 | 0 | 1 | 1 | 1 | 1 |
| *Crepidotus versutus* | 0 | 0 | 0 | 0 | 0 | 1 | 0 | 0 |
| *Cryptocoryneum condensatum* | 0 | 2 | 1 | 0 | 0 | 1 | 0 | 0 |
| *Cyanosporus alni* | 0 | 0 | 0 | 0 | 1 | 1 | 0 | 0 |
| *Cyathicula cyathoidea* | 0 | 0 | 2 | 1 | 0 | 0 | 0 | 0 |
| *Cylindrobasidium laeve* | 38 | 54 | 9 | 38 | 14 | 45 | 3 | 28 |
| *Dacrymyces capitatus* | 13 | 0 | 41 | 0 | 41 | 2 | 31 | 3 |
| *Dacrymyces stillatus* | 7 | 14 | 9 | 34 | 48 | 53 | 24 | 58 |
| *Dacrymyces tortus* | 0 | 0 | 0 | 0 | 1 | 3 | 0 | 0 |
| *Dacryobolus sudans* | 0 | 0 | 0 | 0 | 0 | 5 | 8 | 1 |
| *Dasyscypha nivea* | 0 | 0 | 1 | 0 | 0 | 0 | 0 | 0 |
| *Datronia mollis* | 3 | 19 | 0 | 7 | 0 | 0 | 0 | 1 |
| *Dematioscypha dematiicola* | 3 | 0 | 0 | 0 | 0 | 0 | 0 | 1 |
| *Dentipellis fragilis* | 0 | 6 | 0 | 3 | 0 | 0 | 0 | 1 |
| *Diatrype decorticata* | 23 | 4 | 33 | 8 | 0 | 0 | 0 | 0 |
| *Diatrype disciformis* | 64 | 37 | 31 | 7 | 0 | 0 | 0 | 0 |
| *Diatrype flavovirens* | 3 | 0 | 32 | 1 | 0 | 0 | 0 | 0 |
| *Diatrypella verrucaeformis* | 13 | 0 | 5 | 3 | 0 | 0 | 0 | 0 |
| *Durandiella gallica* | 0 | 0 | 0 | 0 | 8 | 1 | 8 | 0 |
| *Durella macrospora* | 0 | 0 | 0 | 2 | 0 | 0 | 0 | 0 |
| *Echinosphaeria canescens* | 0 | 0 | 1 | 0 | 0 | 0 | 0 | 0 |
| *Entoloma cetratum* | 0 | 0 | 0 | 0 | 1 | 0 | 0 | 0 |
| *Eriosphaeria aggregata* | 0 | 0 | 0 | 0 | 1 | 0 | 0 | 0 |
| *Eutypa spinosa* | 0 | 27 | 0 | 6 | 0 | 0 | 0 | 0 |
| *Eutypella quaternata* | 3 | 8 | 0 | 5 | 0 | 1 | 0 | 0 |
| *Exarmidium inclusum* | 1 | 2 | 14 | 58 | 4 | 4 | 34 | 36 |
| *Exidia pithya* | 0 | 0 | 0 | 1 | 25 | 21 | 1 | 28 |
| *Exidia plana* | 9 | 53 | 1 | 55 | 0 | 1 | 0 | 4 |
| *Exidiopsis calcea* | 0 | 0 | 0 | 0 | 0 | 1 | 0 | 0 |
| *Exidiopsis effusa* | 33 | 11 | 6 | 1 | 0 | 3 | 0 | 0 |
| *Flagelloscypha minutissima* | 0 | 0 | 0 | 0 | 1 | 0 | 0 | 0 |
| *Flammulaster carpophilus* | 0 | 0 | 0 | 0 | 0 | 1 | 0 | 0 |
| *Flammulaster limulata* | 0 | 0 | 1 | 0 | 0 | 0 | 0 | 0 |
| *Flavophlebia sulfureoisabellina* | 0 | 0 | 0 | 0 | 0 | 1 | 0 | 0 |
| *Fomes fomentarius* | 0 | 41 | 0 | 26 | 0 | 1 | 0 | 1 |
| *Fomitopsis pinicola* | 1 | 30 | 0 | 30 | 1 | 28 | 0 | 51 |
| *Fuscopostia fragilis* | 0 | 0 | 0 | 0 | 0 | 5 | 0 | 0 |
| *Galerina camerina* | 0 | 0 | 0 | 0 | 0 | 0 | 0 | 2 |
| *Galerina marginata* | 0 | 2 | 1 | 0 | 0 | 1 | 0 | 2 |
| *Galzinia incrustans* | 0 | 0 | 1 | 0 | 0 | 0 | 0 | 0 |
| *Ganoderma lipsiense* | 0 | 23 | 0 | 15 | 0 | 3 | 0 | 1 |
| *Gloeocystidiellum clavuligerum* | 1 | 1 | 0 | 0 | 0 | 0 | 0 | 0 |
| *Gloeocystidiellum luridum* | 1 | 0 | 0 | 0 | 0 | 0 | 0 | 0 |
| *Gloeocystidiellum porosum* | 1 | 2 | 0 | 0 | 1 | 0 | 0 | 1 |
| *Gloeophyllum odoratum* | 0 | 0 | 0 | 0 | 0 | 1 | 0 | 0 |
| *Gloeophyllum sepiarium* | 0 | 0 | 0 | 7 | 0 | 2 | 92 | 37 |
| *Grandinia aspera* | 1 | 2 | 0 | 2 | 2 | 9 | 0 | 7 |
| *Graphium calicioides* | 0 | 2 | 0 | 0 | 0 | 1 | 0 | 3 |
| *Gymnopilus sapineus* | 0 | 0 | 0 | 2 | 1 | 0 | 0 | 4 |
| *Gymnopus androsaceus* | 0 | 0 | 0 | 0 | 0 | 1 | 6 | 1 |
| *Gyromitra inflata* | 0 | 0 | 0 | 1 | 0 | 0 | 0 | 0 |
| *Hamatocanthoscypha laricionis* | 0 | 1 | 0 | 0 | 1 | 25 | 0 | 11 |
| *Helicogloea farinacea* | 0 | 2 | 0 | 0 | 0 | 1 | 0 | 0 |
| *Helicogloea lagerheimii* | 2 | 3 | 0 | 1 | 0 | 0 | 0 | 0 |
| *Helminthosphaeria odontiae* | 0 | 2 | 0 | 0 | 0 | 0 | 0 | 0 |
| *Helminthosphaeria stuppea* | 0 | 0 | 0 | 0 | 1 | 3 | 0 | 0 |
| *Henningsomyces candidus* | 0 | 0 | 0 | 0 | 2 | 7 | 0 | 0 |
| *Herpotrichia macrotricha* | 0 | 0 | 0 | 0 | 1 | 0 | 0 | 0 |
| *Heterobasidion abietinum* | 0 | 0 | 0 | 0 | 0 | 13 | 0 | 4 |
| *Heterobasidion annosum* | 0 | 0 | 0 | 0 | 0 | 13 | 0 | 6 |
| *Hohenbuehelia atrocoerulea* | 0 | 1 | 0 | 0 | 0 | 1 | 0 | 0 |
| *Hohenbuehelia pinacearum* | 0 | 0 | 0 | 0 | 1 | 0 | 0 | 0 |
| *Hyalorbilia berberidis* | 0 | 0 | 0 | 0 | 1 | 0 | 0 | 0 |
| *Hyalorbilia inflatula* | 11 | 4 | 5 | 2 | 3 | 2 | 0 | 1 |
| *Hyaloscypha albohyalina* | 7 | 0 | 0 | 0 | 0 | 0 | 0 | 0 |
| *Hyaloscypha aureliella* | 6 | 0 | 1 | 0 | 5 | 23 | 4 | 12 |
| *Hyaloscypha britannica* | 0 | 0 | 0 | 0 | 0 | 0 | 1 | 0 |
| *Hyaloscypha spiralis* | 26 | 6 | 7 | 1 | 1 | 1 | 1 | 0 |
| *Hygrophoropsis aurantiaca* | 0 | 0 | 0 | 0 | 0 | 0 | 0 | 1 |
| *Hymenochaete cruenta* | 0 | 0 | 0 | 0 | 1 | 0 | 0 | 0 |
| *Hymenoscyphus caudatus* | 0 | 0 | 0 | 1 | 1 | 0 | 1 | 0 |
| *Hymenoscyphus conscriptus* | 0 | 0 | 0 | 1 | 0 | 0 | 0 | 0 |
| *Hymenoscyphus scutula* | 0 | 0 | 0 | 0 | 0 | 1 | 0 | 0 |
| *Hymenoscyphus virgultorum* | 0 | 10 | 1 | 10 | 0 | 15 | 0 | 5 |
| *Hyphoderma argillaceum* | 4 | 6 | 2 | 1 | 0 | 4 | 0 | 0 |
| *Hyphoderma medioburiense* | 0 | 0 | 0 | 1 | 0 | 0 | 0 | 0 |
| *Hyphoderma mutatum* | 1 | 2 | 0 | 0 | 0 | 0 | 0 | 0 |
| *Hyphoderma obtusiforme* | 1 | 1 | 0 | 0 | 0 | 0 | 0 | 0 |
| *Hyphoderma praetermissum* | 16 | 27 | 5 | 18 | 5 | 19 | 1 | 6 |
| *Hyphoderma puberum* | 5 | 19 | 4 | 31 | 0 | 11 | 1 | 8 |
| *Hyphoderma roseocremeum* | 4 | 2 | 0 | 2 | 0 | 0 | 0 | 0 |
| *Hyphoderma setigerum* | 42 | 35 | 15 | 21 | 8 | 12 | 0 | 3 |
| *Hyphodiscus hemiamyloideus* | 3 | 7 | 0 | 4 | 0 | 0 | 0 | 0 |
| *Hyphodiscus hymeniophila* | 0 | 1 | 0 | 0 | 0 | 0 | 0 | 0 |
| *Hyphodontia alutaria* | 0 | 0 | 0 | 1 | 1 | 0 | 0 | 2 |
| *Hyphodontia arguta* | 0 | 0 | 0 | 1 | 0 | 0 | 0 | 1 |
| *Hyphodontia pallidula* | 0 | 0 | 0 | 4 | 0 | 3 | 0 | 5 |
| *Hypholoma capnoides* | 0 | 1 | 0 | 1 | 0 | 18 | 0 | 6 |
| *Hypholoma fasciculare* | 0 | 11 | 2 | 23 | 0 | 22 | 0 | 14 |
| *Hypholoma marginatum* | 0 | 0 | 0 | 0 | 0 | 0 | 0 | 1 |
| *Hypholoma subviride* | 0 | 1 | 0 | 1 | 0 | 0 | 0 | 0 |
| *Hypochnicium albostramineum* | 0 | 1 | 0 | 1 | 0 | 0 | 0 | 1 |
| *Hypochnicium bombycinum* | 0 | 1 | 0 | 1 | 0 | 0 | 0 | 0 |
| *Hypochnicium cremicolor* | 0 | 0 | 0 | 1 | 0 | 1 | 0 | 1 |
| *Hypochnicium eichleri* | 0 | 0 | 0 | 1 | 0 | 1 | 0 | 0 |
| *Hypochnicium erikssonii* | 0 | 3 | 0 | 2 | 0 | 0 | 0 | 2 |
| *Hypochnicium geogenium* | 0 | 0 | 0 | 0 | 0 | 0 | 0 | 1 |
| *Hypochnicium punctulatum* | 0 | 1 | 0 | 7 | 0 | 3 | 0 | 1 |
| *Hypochnicium subrigescens* | 0 | 6 | 0 | 4 | 0 | 1 | 0 | 3 |
| *Hypochnicium wakefieldiae* | 0 | 0 | 0 | 1 | 0 | 0 | 0 | 0 |
| *Hypocrea aureoviridis* | 0 | 1 | 0 | 0 | 2 | 1 | 0 | 0 |
| *Hypocrea citrina* | 0 | 4 | 0 | 4 | 0 | 2 | 0 | 0 |
| *Hypocrea protopulvinata* | 0 | 0 | 0 | 1 | 0 | 0 | 0 | 3 |
| *Hypocrea rufa* | 35 | 45 | 2 | 5 | 4 | 34 | 0 | 0 |
| *Hypomyces aurantius* | 0 | 0 | 0 | 1 | 0 | 0 | 0 | 0 |
| *Hypoxylon cohaerens* | 90 | 60 | 29 | 60 | 1 | 0 | 0 | 0 |
| *Hypoxylon fragiforme* | 113 | 60 | 79 | 60 | 0 | 0 | 0 | 0 |
| *Hypoxylon howeianum* | 0 | 0 | 0 | 2 | 0 | 0 | 0 | 0 |
| *Hypoxylon rubiginosum* | 44 | 57 | 33 | 41 | 0 | 0 | 0 | 0 |
| *Imleria badia* | 1 | 0 | 0 | 0 | 0 | 0 | 0 | 0 |
| *Inonotus hastifer* | 1 | 1 | 0 | 0 | 0 | 0 | 0 | 0 |
| *Inonotus nodulosus* | 3 | 9 | 1 | 2 | 0 | 0 | 0 | 0 |
| *Irpex lacteus* | 1 | 8 | 5 | 14 | 0 | 2 | 0 | 8 |
| *Ischnoderma benzoinum* | 0 | 0 | 0 | 2 | 0 | 5 | 0 | 3 |
| *Ischnoderma resinosum* | 0 | 0 | 0 | 1 | 0 | 0 | 0 | 0 |
| *Jaapia argillacea* | 0 | 0 | 0 | 0 | 0 | 0 | 0 | 1 |
| *Junghuhnia nitida* | 0 | 0 | 0 | 0 | 0 | 0 | 0 | 1 |
| *Kneiffia subalutacea* | 1 | 1 | 0 | 0 | 0 | 1 | 1 | 1 |
| *Kneiffiella abieticola* | 0 | 0 | 0 | 1 | 0 | 0 | 0 | 0 |
| *Kneiffiella barba-jovis* | 0 | 0 | 0 | 1 | 0 | 0 | 0 | 0 |
| *Kneiffiella microspora* | 1 | 0 | 0 | 0 | 0 | 0 | 0 | 0 |
| *Kretzschmaria deusta* | 1 | 9 | 0 | 1 | 0 | 0 | 0 | 0 |
| *Kuehneromyces mutabilis* | 0 | 3 | 0 | 0 | 0 | 3 | 0 | 0 |
| *Kurtia macedonica* | 2 | 0 | 0 | 0 | 0 | 0 | 0 | 0 |
| *Laccaria amethystea* | 2 | 1 | 0 | 0 | 2 | 6 | 0 | 0 |
| *Laccaria laccata* | 0 | 0 | 0 | 1 | 0 | 0 | 0 | 0 |
| *Lachnellula abietis* | 0 | 0 | 0 | 0 | 2 | 0 | 1 | 0 |
| *Lachnellula calyciformis* | 0 | 0 | 0 | 0 | 47 | 1 | 12 | 2 |
| *Lachnellula gallica* | 0 | 0 | 0 | 0 | 16 | 0 | 28 | 0 |
| *Lachnellula subtilissima* | 0 | 0 | 0 | 0 | 0 | 0 | 1 | 0 |
| *Lachnum fasciculare* | 1 | 0 | 0 | 0 | 0 | 0 | 0 | 0 |
| *Lachnum impudicum* | 1 | 0 | 0 | 0 | 0 | 0 | 0 | 0 |
| *Lachnum virgineum* | 7 | 1 | 20 | 1 | 0 | 0 | 0 | 0 |
| *Lactarius camphoratus* | 0 | 0 | 0 | 0 | 0 | 1 | 0 | 0 |
| *Lactarius helvus* | 1 | 1 | 0 | 0 | 1 | 1 | 0 | 0 |
| *Lactarius subdulcis* | 0 | 1 | 0 | 0 | 0 | 0 | 0 | 0 |
| *Lasiosphaeria canescens* | 0 | 0 | 4 | 1 | 1 | 4 | 0 | 1 |
| *Lasiosphaeria hirsuta* | 1 | 4 | 0 | 2 | 0 | 1 | 0 | 0 |
| *Lasiosphaeria ovina* | 2 | 12 | 0 | 5 | 0 | 0 | 0 | 0 |
| *Lasiosphaeria spermoides* | 1 | 6 | 1 | 5 | 0 | 1 | 0 | 0 |
| *Lasiosphaeria strigosa* | 3 | 3 | 7 | 3 | 3 | 23 | 0 | 6 |
| *Lawrynomyces capitatus* | 0 | 0 | 0 | 0 | 0 | 0 | 0 | 1 |
| *Laxitextum bicolor* | 19 | 17 | 5 | 10 | 1 | 1 | 0 | 0 |
| *Lentomitella cirrhosa* | 0 | 0 | 0 | 1 | 0 | 0 | 0 | 0 |
| *Lenzites betulinus* | 3 | 2 | 9 | 28 | 0 | 0 | 0 | 1 |
| *Leptodontidium trabinellum* | 4 | 4 | 0 | 0 | 0 | 0 | 0 | 0 |
| *Leptosporomyces galzinii* | 0 | 0 | 0 | 0 | 2 | 0 | 0 | 0 |
| *Leptosporomyces mutabilis* | 0 | 0 | 0 | 0 | 0 | 2 | 0 | 0 |
| *Leptosporomyces roseus* | 3 | 0 | 0 | 0 | 6 | 6 | 0 | 3 |
| *Leucoscypha leucotricha* | 1 | 0 | 0 | 0 | 0 | 3 | 0 | 0 |
| *Lopadostoma turgidum* | 7 | 0 | 2 | 0 | 0 | 0 | 0 | 0 |
| *Lopharia spadicea* | 0 | 1 | 4 | 2 | 1 | 0 | 0 | 0 |
| *Lophiotrema boreale* | 0 | 9 | 0 | 0 | 0 | 1 | 0 | 0 |
| *Lophium mytilinum* | 0 | 0 | 0 | 0 | 54 | 44 | 42 | 10 |
| *Lycoperdon perlatum* | 0 | 0 | 0 | 1 | 0 | 0 | 0 | 0 |
| *Lyomyces crustosus* | 0 | 0 | 2 | 0 | 0 | 2 | 0 | 0 |
| *Lyomyces sambuci* | 0 | 0 | 0 | 0 | 0 | 1 | 0 | 0 |
| *Melanomma pulvispyrius* | 33 | 59 | 30 | 24 | 2 | 20 | 1 | 4 |
| *Melanomma sanguinarium* | 0 | 1 | 0 | 0 | 0 | 0 | 0 | 0 |
| *Melanotus phillipsii* | 0 | 0 | 0 | 1 | 0 | 0 | 0 | 0 |
| *Merismodes anomalus* | 4 | 29 | 0 | 19 | 0 | 0 | 0 | 0 |
| *Metulodontia nivea* | 1 | 0 | 0 | 0 | 0 | 0 | 0 | 0 |
| *Mollisia aquosa* | 0 | 6 | 0 | 6 | 0 | 1 | 0 | 1 |
| *Mollisia fusca* | 7 | 0 | 4 | 3 | 0 | 0 | 0 | 0 |
| *Mollisia ligni* | 1 | 1 | 1 | 1 | 0 | 0 | 0 | 0 |
| *Mollisia lividofusca* | 1 | 4 | 7 | 7 | 0 | 0 | 0 | 0 |
| *Mollisia olivaceocinerea* | 100 | 60 | 110 | 54 | 1 | 9 | 2 | 6 |
| *Mucronella calva* | 0 | 2 | 0 | 0 | 0 | 9 | 0 | 6 |
| *Mycena abramsii* | 1 | 0 | 0 | 0 | 0 | 0 | 0 | 0 |
| *Mycena galericulata* | 0 | 1 | 0 | 3 | 0 | 0 | 0 | 0 |
| *Mycena galopus* | 1 | 0 | 0 | 1 | 0 | 0 | 0 | 1 |
| *Mycena haematopus* | 3 | 8 | 0 | 8 | 0 | 6 | 0 | 0 |
| *Mycena leptocephala* | 0 | 0 | 0 | 1 | 0 | 1 | 0 | 0 |
| *Mycena metata* | 0 | 0 | 0 | 0 | 2 | 0 | 0 | 0 |
| *Mycena pura* | 0 | 0 | 0 | 0 | 0 | 1 | 0 | 0 |
| *Mycena renati* | 0 | 3 | 0 | 0 | 0 | 0 | 0 | 0 |
| *Mycena rubromarginata* | 1 | 1 | 1 | 3 | 0 | 1 | 0 | 1 |
| *Mycena sanguinolenta* | 1 | 1 | 0 | 2 | 0 | 0 | 0 | 1 |
| *Mycena zephirus* | 0 | 0 | 0 | 0 | 0 | 1 | 0 | 0 |
| *Mycetinis alliaceus* | 0 | 1 | 0 | 0 | 0 | 0 | 0 | 0 |
| *Mycoacia nothofagi* | 0 | 2 | 0 | 2 | 0 | 0 | 0 | 0 |
| *Myxarium grilletii* | 0 | 3 | 2 | 1 | 0 | 0 | 0 | 0 |
| *Natantiella ligneola* | 0 | 1 | 2 | 1 | 0 | 4 | 1 | 3 |
| *Nectria cinnabarina* | 0 | 8 | 0 | 2 | 0 | 0 | 0 | 0 |
| *Nectria coccinea* | 38 | 58 | 15 | 23 | 3 | 3 | 0 | 0 |
| *Nectria cosmariospora* | 0 | 1 | 0 | 0 | 0 | 0 | 0 | 0 |
| *Nectria episphaeria* | 12 | 1 | 3 | 0 | 0 | 0 | 0 | 0 |
| *Nectria fuckeliana* | 0 | 0 | 0 | 0 | 25 | 28 | 7 | 4 |
| *Nectria magnusiana* | 2 | 1 | 1 | 0 | 0 | 0 | 0 | 0 |
| *Nectria peziza* | 0 | 0 | 0 | 0 | 0 | 1 | 0 | 1 |
| *Nemania chestersii* | 0 | 1 | 0 | 0 | 0 | 0 | 0 | 0 |
| *Nemania serpens* | 3 | 11 | 1 | 0 | 0 | 0 | 0 | 0 |
| *Nematogonum ferrugineum* | 0 | 1 | 0 | 2 | 0 | 4 | 0 | 0 |
| *Neobulgaria pura* | 43 | 30 | 3 | 5 | 0 | 0 | 0 | 0 |
| *Neodasyscypha cerina* | 7 | 58 | 4 | 41 | 0 | 0 | 0 | 0 |
| *Nidularia deformis* | 0 | 0 | 1 | 0 | 0 | 0 | 0 | 0 |
| *Olla scropulosa* | 1 | 0 | 0 | 0 | 0 | 0 | 0 | 0 |
| *Olla transiens* | 1 | 0 | 0 | 0 | 0 | 0 | 0 | 0 |
| *Ombrophila disciformis* | 2 | 5 | 1 | 4 | 1 | 0 | 0 | 0 |
| *Ombrophila janthina* | 0 | 2 | 0 | 4 | 0 | 0 | 0 | 0 |
| *Ombrophila violacea* | 1 | 1 | 0 | 0 | 0 | 0 | 0 | 0 |
| *Orbilia coccinella* | 1 | 2 | 0 | 0 | 0 | 0 | 0 | 0 |
| *Orbilia delicatula* | 61 | 59 | 11 | 59 | 5 | 48 | 2 | 28 |
| *Orbilia leucostigma* | 0 | 0 | 0 | 0 | 1 | 1 | 0 | 0 |
| *Orbilia sarraziniana* | 2 | 2 | 0 | 1 | 0 | 0 | 0 | 0 |
| *Oudemansiella mucida* | 0 | 6 | 0 | 3 | 0 | 0 | 0 | 0 |
| *Panellus mitis* | 0 | 0 | 0 | 1 | 6 | 18 | 1 | 5 |
| *Panellus serotinus* | 0 | 6 | 1 | 7 | 0 | 0 | 0 | 0 |
| *Panellus stypticus* | 0 | 11 | 0 | 4 | 0 | 0 | 0 | 0 |
| *Panellus violaceofulvus* | 1 | 1 | 0 | 0 | 5 | 27 | 0 | 5 |
| *Parorbiliopsis minuta* | 4 | 8 | 0 | 2 | 0 | 3 | 0 | 1 |
| *Patinella sanguineoatra* | 0 | 1 | 0 | 2 | 0 | 0 | 0 | 0 |
| *Paullicorticium pearsonii* | 0 | 0 | 0 | 0 | 1 | 0 | 0 | 0 |
| *Pellidiscus pallidus* | 0 | 0 | 0 | 0 | 5 | 0 | 0 | 0 |
| *Peniophora cinerea* | 11 | 25 | 20 | 46 | 0 | 2 | 1 | 3 |
| *Peniophora incarnata* | 3 | 8 | 1 | 15 | 0 | 4 | 0 | 1 |
| *Peniophora piceae* | 0 | 0 | 0 | 2 | 3 | 1 | 1 | 4 |
| *Peniophora pithya* | 0 | 0 | 0 | 1 | 6 | 5 | 17 | 1 |
| *Peniophora violaceolivida* | 0 | 0 | 0 | 1 | 0 | 0 | 0 | 0 |
| *Peniophorella pallida* | 1 | 0 | 1 | 0 | 0 | 3 | 0 | 1 |
| *Peniophorella tsugae* | 0 | 2 | 0 | 1 | 0 | 0 | 0 | 0 |
| *Pezicula acericola* | 1 | 4 | 1 | 0 | 0 | 1 | 0 | 0 |
| *Pezicula cinnamomea* | 0 | 0 | 1 | 0 | 0 | 0 | 0 | 0 |
| *Phaeohelotium carneum* | 2 | 0 | 0 | 0 | 0 | 1 | 0 | 0 |
| *Phaeohelotium trabinellum* | 0 | 1 | 0 | 0 | 0 | 0 | 0 | 0 |
| *Phanerochaete filamentosa* | 0 | 0 | 1 | 0 | 0 | 0 | 0 | 0 |
| *Phanerochaete galactites* | 1 | 1 | 1 | 0 | 0 | 0 | 1 | 0 |
| *Phanerochaete laevis* | 22 | 5 | 1 | 0 | 7 | 1 | 0 | 0 |
| *Phanerochaete leprosa* | 0 | 1 | 0 | 1 | 0 | 0 | 0 | 0 |
| *Phanerochaete livescens* | 32 | 9 | 1 | 1 | 1 | 1 | 0 | 0 |
| *Phanerochaete raduloides* | 0 | 0 | 1 | 0 | 0 | 0 | 0 | 0 |
| *Phanerochaete sanguinea* | 2 | 0 | 1 | 0 | 0 | 0 | 0 | 0 |
| *Phanerochaete sordida* | 21 | 5 | 11 | 2 | 30 | 7 | 4 | 1 |
| *Phanerochaete tuberculata* | 1 | 0 | 3 | 0 | 0 | 0 | 0 | 0 |
| *Phanerochaete velutina* | 17 | 13 | 0 | 1 | 1 | 2 | 0 | 0 |
| *Phellinus ferruginosus* | 0 | 0 | 1 | 0 | 0 | 0 | 0 | 0 |
| *Phellinus hartigii* | 0 | 0 | 0 | 0 | 0 | 1 | 0 | 0 |
| *Phlebia acerina* | 1 | 0 | 0 | 0 | 0 | 0 | 0 | 0 |
| *Phlebia lilascens* | 0 | 1 | 0 | 2 | 2 | 2 | 0 | 1 |
| *Phlebia livida* | 0 | 3 | 0 | 4 | 0 | 0 | 0 | 0 |
| *Phlebia queletii* | 0 | 0 | 0 | 0 | 0 | 0 | 0 | 1 |
| *Phlebia radiata* | 14 | 29 | 11 | 18 | 0 | 2 | 0 | 2 |
| *Phlebia rufa* | 12 | 13 | 2 | 8 | 0 | 0 | 0 | 0 |
| *Phlebia tremellosa* | 3 | 1 | 6 | 6 | 0 | 1 | 0 | 5 |
| *Phlebiella tulasnelloidea* | 0 | 3 | 1 | 0 | 0 | 2 | 0 | 0 |
| *Phlebiella vaga* | 28 | 10 | 5 | 1 | 7 | 13 | 6 | 2 |
| *Phlebiopsis gigantea* | 0 | 0 | 0 | 0 | 1 | 3 | 0 | 10 |
| *Pholiota cerifera* | 0 | 0 | 0 | 2 | 0 | 1 | 0 | 0 |
| *Pholiota lenta* | 0 | 1 | 0 | 1 | 0 | 3 | 0 | 0 |
| *Pholiota limonella* | 0 | 1 | 0 | 0 | 0 | 0 | 0 | 0 |
| *Pholiota tuberculosa* | 0 | 1 | 1 | 3 | 0 | 0 | 0 | 0 |
| *Physisporinus sanguinolentus* | 1 | 0 | 1 | 7 | 0 | 1 | 0 | 9 |
| *Physisporinus vitreus* | 0 | 0 | 0 | 1 | 0 | 0 | 0 | 0 |
| *Pleurotus ostreatus* | 0 | 5 | 0 | 6 | 0 | 1 | 0 | 0 |
| *Pleurotus pulmonarius* | 0 | 8 | 0 | 1 | 0 | 0 | 0 | 0 |
| *Plicaturopsis crispa* | 4 | 4 | 2 | 4 | 0 | 1 | 0 | 0 |
| *Pluteus cervinus* | 2 | 4 | 0 | 14 | 0 | 2 | 0 | 0 |
| *Pluteus semibulbosus* | 0 | 0 | 0 | 1 | 0 | 0 | 0 | 0 |
| *Polydesmia pruinosa* | 2 | 11 | 0 | 0 | 0 | 1 | 0 | 0 |
| *Polyporus brumalis* | 2 | 1 | 36 | 11 | 0 | 0 | 1 | 1 |
| *Polyporus ciliatus* | 0 | 0 | 8 | 1 | 0 | 0 | 0 | 0 |
| *Polyporus varius* | 1 | 1 | 0 | 2 | 0 | 0 | 0 | 0 |
| *Postia caesia* | 0 | 1 | 0 | 3 | 6 | 39 | 1 | 14 |
| *Postia guttulata* | 0 | 0 | 0 | 1 | 0 | 0 | 0 | 0 |
| *Postia stiptica* | 0 | 0 | 0 | 3 | 0 | 1 | 0 | 0 |
| *Postia subcaesia* | 0 | 11 | 1 | 2 | 1 | 0 | 0 | 0 |
| *Postia tephroleuca* | 0 | 24 | 1 | 19 | 0 | 3 | 0 | 4 |
| *Postia undosa* | 0 | 1 | 0 | 1 | 0 | 2 | 0 | 0 |
| *Proliferodiscus pulveraceus* | 0 | 1 | 0 | 0 | 0 | 0 | 0 | 0 |
| *Psathyrella obtusata* | 0 | 0 | 0 | 0 | 0 | 1 | 0 | 0 |
| *Psathyrella piluliformis* | 0 | 1 | 0 | 0 | 0 | 0 | 0 | 0 |
| *Pseudohydnum gelatinosum* | 0 | 0 | 0 | 0 | 0 | 2 | 0 | 0 |
| *Pseudotomentella tristis* | 1 | 0 | 0 | 0 | 0 | 0 | 0 | 0 |
| *Pseudotomentella umbrina* | 1 | 0 | 0 | 0 | 0 | 0 | 0 | 0 |
| *Psilocistella quercina* | 25 | 6 | 7 | 3 | 0 | 0 | 1 | 1 |
| *Psilocybe phyllogena* | 0 | 0 | 1 | 0 | 0 | 0 | 0 | 0 |
| *Pycnoporellus fulgens* | 0 | 2 | 0 | 2 | 0 | 6 | 0 | 30 |
| *Pycnoporus cinnabarinus* | 1 | 0 | 40 | 32 | 0 | 0 | 1 | 4 |
| *Radulomyces confluens* | 3 | 1 | 2 | 2 | 4 | 2 | 1 | 1 |
| *Resinicium bicolor* | 1 | 1 | 0 | 9 | 0 | 13 | 1 | 19 |
| *Resupinatus applicatus* | 0 | 0 | 2 | 2 | 0 | 0 | 0 | 0 |
| *Resupinatus trichotis* | 0 | 1 | 1 | 1 | 0 | 0 | 0 | 0 |
| *Rhodocollybia butyracea* | 0 | 1 | 0 | 0 | 0 | 0 | 0 | 0 |
| *Rigidoporus sanguinolentus* | 0 | 0 | 1 | 0 | 0 | 0 | 0 | 1 |
| *Rigidoporus vitreus* | 0 | 1 | 0 | 0 | 0 | 0 | 0 | 5 |
| *Rosellinia aquila* | 0 | 1 | 0 | 0 | 0 | 4 | 0 | 1 |
| *Rosellinia thelena* | 0 | 3 | 0 | 1 | 4 | 13 | 0 | 9 |
| *Russula mairei* | 0 | 0 | 0 | 0 | 0 | 4 | 0 | 0 |
| *Schizophyllum commune* | 9 | 1 | 74 | 57 | 0 | 1 | 19 | 20 |
| *Schizopora flavipora* | 0 | 0 | 0 | 0 | 0 | 2 | 0 | 1 |
| *Schizopora paradoxa* | 5 | 29 | 2 | 12 | 1 | 10 | 0 | 8 |
| *Schizopora radula* | 0 | 0 | 0 | 0 | 0 | 3 | 0 | 1 |
| *Scopuloides rimosa* | 0 | 0 | 0 | 2 | 0 | 0 | 0 | 0 |
| *Scutellinia cejpii* | 0 | 0 | 0 | 0 | 0 | 3 | 0 | 1 |
| *Scutellinia scutellata* | 0 | 1 | 0 | 0 | 0 | 2 | 0 | 0 |
| *Scutellinia subhirtella* | 0 | 1 | 0 | 0 | 0 | 3 | 0 | 0 |
| *Scutellinia umbrorum* | 0 | 0 | 0 | 0 | 0 | 2 | 0 | 1 |
| *Sebacina grisea* | 0 | 4 | 2 | 0 | 6 | 30 | 1 | 9 |
| *Sebacina incrustans* | 1 | 0 | 0 | 1 | 0 | 0 | 0 | 0 |
| *Serpula himantioides* | 0 | 0 | 0 | 0 | 0 | 2 | 0 | 1 |
| *Simocybe centunculus* | 0 | 6 | 1 | 2 | 0 | 0 | 0 | 0 |
| *Simocybe coniophora* | 0 | 1 | 0 | 0 | 0 | 0 | 0 | 0 |
| *Simocybe haustellaris* | 0 | 0 | 0 | 2 | 0 | 0 | 0 | 0 |
| *Sistotrema brinkmannii* | 27 | 25 | 38 | 23 | 9 | 21 | 4 | 21 |
| *Sistotrema confluens* | 0 | 0 | 0 | 0 | 0 | 0 | 0 | 1 |
| *Sistotrema coroniferum* | 0 | 0 | 0 | 0 | 1 | 0 | 0 | 0 |
| *Sistotrema diademiferum* | 0 | 0 | 2 | 0 | 0 | 1 | 0 | 0 |
| *Sistotrema efibulatum* | 0 | 0 | 0 | 0 | 1 | 0 | 0 | 0 |
| *Sistotrema oblongisporum* | 0 | 1 | 1 | 0 | 0 | 0 | 0 | 0 |
| *Sistotrema octosporum* | 1 | 0 | 3 | 0 | 0 | 0 | 1 | 2 |
| *Sistotrema porulosum* | 0 | 1 | 0 | 0 | 0 | 0 | 0 | 0 |
| *Sistotrema sernanderi* | 0 | 0 | 1 | 1 | 0 | 0 | 0 | 0 |
| *Sistotremastrum niveocremeum* | 3 | 0 | 5 | 0 | 0 | 1 | 0 | 0 |
| *Sistotremastrum suecicum* | 0 | 0 | 0 | 0 | 0 | 2 | 0 | 2 |
| *Skeletocutis amorpha* | 0 | 0 | 0 | 0 | 0 | 0 | 0 | 1 |
| *Skeletocutis carneogrisea* | 0 | 0 | 2 | 1 | 0 | 0 | 3 | 1 |
| *Skeletocutis nivea* | 1 | 2 | 2 | 3 | 0 | 0 | 0 | 0 |
| *Skvortzovia furfuracea* | 0 | 0 | 0 | 0 | 0 | 0 | 0 | 2 |
| *Sphaerobolus stellatus* | 0 | 0 | 7 | 0 | 0 | 0 | 2 | 0 |
| *Steccherinum ochraceum* | 0 | 3 | 0 | 0 | 0 | 0 | 0 | 2 |
| *Stereum hirsutum* | 36 | 56 | 18 | 53 | 0 | 0 | 0 | 0 |
| *Stereum rameale* | 0 | 0 | 1 | 0 | 0 | 0 | 0 | 0 |
| *Stereum rugosum* | 18 | 38 | 0 | 17 | 0 | 1 | 0 | 1 |
| *Stereum sanguinolentum* | 0 | 0 | 0 | 0 | 88 | 56 | 14 | 52 |
| *Stereum subtomentosum* | 2 | 2 | 0 | 0 | 0 | 0 | 0 | 0 |
| *Strossmayeria basitricha* | 4 | 0 | 0 | 0 | 0 | 0 | 0 | 0 |
| *Tapinella panuoides* | 0 | 0 | 0 | 0 | 0 | 1 | 0 | 0 |
| *Thelephora atra* | 0 | 0 | 0 | 0 | 0 | 0 | 0 | 1 |
| *Thelephora terrestris* | 68 | 19 | 30 | 22 | 58 | 33 | 29 | 28 |
| *Tomentella botryoides* | 2 | 2 | 0 | 0 | 0 | 0 | 0 | 0 |
| *Tomentella cinerascens* | 0 | 0 | 0 | 1 | 0 | 0 | 0 | 0 |
| *Tomentella lilacinogrisea* | 0 | 0 | 0 | 0 | 1 | 1 | 0 | 0 |
| *Tomentella sublilacina* | 0 | 0 | 0 | 1 | 0 | 1 | 0 | 0 |
| *Tomentella testaceogilva* | 0 | 1 | 0 | 0 | 0 | 0 | 0 | 0 |
| *Trametes gibbosa* | 0 | 21 | 0 | 27 | 0 | 0 | 0 | 1 |
| *Trametes hirsuta* | 11 | 11 | 83 | 59 | 0 | 1 | 3 | 12 |
| *Trametes multicolor* | 0 | 0 | 0 | 1 | 0 | 0 | 0 | 0 |
| *Trametes ochracea* | 0 | 3 | 0 | 4 | 0 | 0 | 0 | 0 |
| *Trametes versicolor* | 5 | 33 | 25 | 56 | 0 | 2 | 1 | 11 |
| *Trechispora cohaerens* | 0 | 0 | 0 | 1 | 0 | 1 | 0 | 0 |
| *Trechispora farinacea* | 0 | 3 | 1 | 2 | 1 | 0 | 1 | 1 |
| *Trechispora hymenocystis* | 6 | 1 | 0 | 3 | 1 | 6 | 0 | 7 |
| *Trechispora microspora* | 0 | 0 | 0 | 0 | 0 | 0 | 1 | 0 |
| *Trechispora minima* | 0 | 0 | 0 | 0 | 0 | 0 | 1 | 0 |
| *Trechispora mollusca* | 4 | 0 | 0 | 1 | 0 | 2 | 0 | 2 |
| *Trechispora nivea* | 0 | 0 | 0 | 0 | 0 | 0 | 1 | 0 |
| *Tremella encephala* | 0 | 0 | 0 | 0 | 3 | 0 | 0 | 0 |
| *Tremella foliacea* | 3 | 7 | 1 | 0 | 0 | 0 | 0 | 2 |
| *Tremella obscura* | 0 | 0 | 0 | 0 | 0 | 0 | 1 | 0 |
| *Trichaptum abietinum* | 0 | 0 | 0 | 0 | 2 | 5 | 1 | 10 |
| *Trichaptum biforme* | 0 | 0 | 0 | 1 | 0 | 0 | 0 | 0 |
| *Tricholomopsis decora* | 0 | 0 | 0 | 2 | 0 | 0 | 0 | 2 |
| *Tricholomopsis rutilans* | 0 | 0 | 0 | 0 | 0 | 2 | 0 | 0 |
| *Trichophaea pseudogregaria* | 0 | 0 | 0 | 0 | 0 | 0 | 0 | 1 |
| *Tromeropsis microtheca* | 0 | 4 | 6 | 22 | 0 | 4 | 21 | 52 |
| *Tubeufia cerea* | 1 | 2 | 6 | 0 | 0 | 0 | 0 | 0 |
| *Tubulicrinis strangulatus* | 0 | 0 | 0 | 0 | 0 | 0 | 0 | 1 |
| *Tubulicrinis subulatus* | 0 | 0 | 0 | 0 | 0 | 0 | 0 | 1 |
| *Tulasnella eichleriana* | 3 | 5 | 2 | 1 | 0 | 5 | 0 | 1 |
| *Tulasnella inclusa* | 0 | 0 | 1 | 0 | 0 | 0 | 0 | 0 |
| *Tulasnella pinicola* | 0 | 1 | 0 | 0 | 0 | 0 | 0 | 0 |
| *Tulasnella pruinosa* | 3 | 0 | 0 | 0 | 0 | 0 | 0 | 0 |
| *Tulasnella thelephorea* | 1 | 0 | 0 | 0 | 0 | 0 | 0 | 0 |
| *Tulasnella violacea* | 0 | 1 | 1 | 0 | 0 | 0 | 0 | 0 |
| *Tulasnella violea* | 5 | 12 | 6 | 1 | 0 | 1 | 1 | 0 |
| *Tylospora asterophora* | 0 | 0 | 0 | 0 | 0 | 0 | 0 | 1 |
| *Tylospora fibrillosa* | 2 | 1 | 0 | 0 | 2 | 2 | 0 | 0 |
| *Tympanis hypopodia* | 0 | 0 | 0 | 0 | 0 | 1 | 1 | 2 |
| *Typhula erythropus* | 0 | 2 | 0 | 1 | 0 | 2 | 0 | 1 |
| *Typhula setipes* | 0 | 0 | 0 | 0 | 0 | 6 | 0 | 5 |
| *Unguicularia cirrhata* | 0 | 2 | 0 | 0 | 0 | 0 | 0 | 0 |
| *Valsaria insitiva* | 0 | 0 | 1 | 13 | 0 | 0 | 0 | 0 |
| *Xenasmatella ardosiaca* | 0 | 1 | 0 | 0 | 0 | 0 | 0 | 0 |
| *Xenasmatella fibrillosa* | 0 | 0 | 0 | 1 | 0 | 0 | 0 | 0 |
| *Xylaria hypoxylon* | 4 | 28 | 0 | 5 | 0 | 0 | 0 | 0 |
| *Xylaria polymorpha* | 0 | 2 | 0 | 0 | 0 | 0 | 0 | 0 |
| *Xylodon brevisetus* | 0 | 0 | 2 | 0 | 1 | 5 | 0 | 6 |
| *Xylodon nespori* | 0 | 2 | 1 | 1 | 1 | 8 | 0 | 1 |
| *Xylodon rimosissimus* | 0 | 0 | 0 | 1 | 0 | 0 | 0 | 0 |
| *Xylodon spathulatus* | 0 | 8 | 0 | 6 | 0 | 20 | 0 | 13 |

**Table S2 Statistics table for treatment-based alpha diversity in response to decay stage and the canopy treatment for dead wood logs and branches.** We fit linear models with treatment-based alpha diversity as response and factorial canopy treatment, continuous decay stage and their interaction term as predictors. Alpha level is 0.016 (Bonferroni adjustment due to multiple comparisons). P values for fixed effects are not displayed due to repeated testing. The abbreviations stand for: t= t-value, edf= effective degrees of freedom, p= p-value.

|  |  | **Logs** | | | **Branches** | | |
| --- | --- | --- | --- | --- | --- | --- | --- |
| **q** | **Predictor** | **t** | **p** | **R^2^** | **t** | **p** | **R^2^** |
| 0 rare | Intercept | 5,38 |  | 0,49 | 6,52 |  | 0,28 |
|  | Canopy - open vs. closed | 0,20 |  |  | -0,53 |  |  |
|  | Time | -3,24 |  |  | -2,27 |  |  |
|  | Time x Canopy | -0,25 | 0,811 |  | -0,39 | 0,712 |  |
| 1 common | Intercept | 6,75 |  | 0,31 | 6,40 |  | 0,06 |
|  | Canopy - open vs. closed | -0,46 |  |  | -0,74 |  |  |
|  | Time | -2,40 |  |  | -0,99 |  |  |
|  | Time x Canopy | -0,32 | 0,762 |  | -0,18 | 0,865 |  |
| 2 dominant | Intercept | 7,64 |  | 0,39 | 7,79 |  | 0,35 |
|  | Canopy - open vs. closed | -1,31 |  |  | -1,47 |  |  |
|  | Time | -2,46 |  |  | -2,19 |  |  |
|  | Time x Canopy | -0,51 | 0,627 |  | -0,83 | 0,440 |  |
| 0 rare | Intercept | 5,05 |  | 0,45 | 4,60 |  | 0,38 |
|  | Canopy - open vs. closed | 0,06 |  |  | -0,94 |  |  |
|  | Time | -3,07 |  |  | -2,56 |  |  |
|  | Time x Canopy | 1,45 | 0,198 |  | -0,32 | 0,760 |  |
| 1 common | Intercept | 5,85 |  | 0,39 | 5,81 |  | 0,54 |
|  | Canopy - open vs. closed | -0,55 |  |  | -3,25 |  |  |
|  | Time | -2,71 |  |  | -1,49 |  |  |
|  | Time x Canopy | 1,09 | 0,319 |  | -0,42 | 0,692 |  |
| 2 dominant | Intercept | 6,63 |  | 0,43 | 3,85 |  | 0,38 |
|  | Canopy - open vs. closed | -1,76 |  |  | -2,59 |  |  |
|  | Time | -2,39 |  |  | -0,91 |  |  |
|  | Time x Canopy | -0,07 | 0,944 |  | -0,32 | 0,762 |  |

**Table S3 Statistics table for beta diversity in response to decay stages.** We fit generalized additive models (GAM) and linear models. Using Tukey post-hoc test we tested for pairwise differences in means between diversity measures. Alpha level is 0.016 (Bonferroni adjustment due to multiple comparisons). The abbreviations stand for: edf= effective degrees of freedom, F=F-value, p= p-value.

|  |  |  |  |  |  | **Tukey pairwise means** | | | |
| --- | --- | --- | --- | --- | --- | --- | --- | --- | --- |
| **Predictor** | **Tree** | **q** | **edf** | **F** | **p** | **R^2^** | **Pair** | **t** | **p** |
| Decay stage | Beech log | 0, rare | 1.00 | 5.92 | 0.033 | 0.24 | 0-1 | 1.88 | 0.185 |
|  |  | 1, common | 1.00 | 1.51 | 0.245 |  | 0-2 | 0.59 | 0.829 |
|  |  | 2, dominant | 1.00 | 0.00 | 0.989 |  | 1-2 | -1.30 | 0.424 |
|  | Fir log | 0, rare | 1.00 | 3.18 | 0.105 | 0.05 | 0-1 | 1.66 | 0.259 |
|  |  | 1, common | 1.00 | 1.27 | 0.285 |  | 0-2 | 2.00 | 0.155 |
|  |  | 2, dominant | 1.52 | 0.41 | 0.596 |  | 1-2 | 0.34 | 0.940 |
| Decay stage | Beech branch | 0, rare | 1.00 | 1.24 | 0.275 | 0.24 | 0-1 | **6.22** | **0.002** |
|  |  | 1, common | 1.00 | 4.76 | 0.038 |  | 0-2 | **6.04** | **0.002** |
|  |  | 2, dominant | 1.46 | **8.37** | **0.005** |  | 1-2 | -0.18 | 0.983 |
|  | Fir branch | 0, rare | 1.00 | 3.84 | 0.107 | 0.05 | 0-1 | 0.93 | 0.640 |
|  |  | 1, common | 1.00 | 2.86 | 0.152 |  | 0-2 | 1.30 | 0.445 |
|  |  | 2, dominant | 1.00 | 4.19 | 0.096 |  | 1-2 | 0.37 | 0.930 |

**References**

Albrecht, L. (1990). *Grundlagen, Ziele und Methodik der waldökologischen Forschung in Naturwaldreservaten*. Bayerisches Staatsministerium für Ernährung, Landwirtschaft und Forsten.
